# The ROX index as a predictor of high-flow nasal cannula outcome in pneumonia patients with acute hypoxemic respiratory failure: a systematic review and meta-analysis

**DOI:** 10.1186/s12890-022-01914-2

**Published:** 2022-04-01

**Authors:** Xiaoyang Zhou, Jiequan Liu, Jianneng Pan, Zhaojun Xu, Jianfei Xu

**Affiliations:** 1 Department of Intensive Care Medicine, HwaMei Hospital, University of Chinese Academy of Sciences, Ningbo, 315000 Zhejiang China; 2 Ningbo Institute of Life and Health Industry, University of Chinese Academy of Sciences, Ningbo, 315000 Zhejiang China; 3 Department of Emergency, Ningbo Yinzhou No.2 Hospital, Ningbo, 315000 Zhejiang China

**Keywords:** High flow nasal cannula, ROX index, Pneumonia, Acute respiratory failure, Intubation

## Abstract

**Background:**

The respiratory rate-oxygenation (ROX) index has been increasingly applied to predict the outcome of high-flow nasal cannula (HFNC) in pneumonia patients with acute hypoxemic respiratory failure (AHRF). However, its diagnostic accuracy for the HFNC outcome has not yet been systematically assessed. This meta-analysis sought to evaluate the predictive performance of the ROC index for the successful weaning from HFNC in pneumonia patients with AHRF.

**Methods:**

A literature search was conducted on electronic databases through February 12, 2022, to retrieve studies that investigated the diagnostic accuracy of the ROC index for the outcome of HFNC application in pneumonia patients with AHRF. The area under the hierarchical summary receiver operating characteristic curve (AUHSROC) was estimated as the primary measure of diagnostic accuracy due to the varied cutoff values of the index. We observed the distribution of the cutoff values and estimated the optimal threshold with corresponding 95% confidential interval (CI).

**Results:**

Thirteen observational studies comprising 1751 patients were included, of whom 1003 (57.3%) successfully weaned from HFNC. The ROC index exhibits good performance for predicting the successful weaning from HFNC in pneumonia patients with AHRF, with an AUHSROC of 0.81 (95% CI 0.77–0.84), a pooled sensitivity of 0.71 (95% CI 0.64–0.78), and a pooled specificity of 0.78 (95% CI 0.70–0.84). The cutoff values of the ROX index were nearly conically symmetrically distributed; most data were centered between 4.5 and 6.0, and the mean and median values were 4.8 (95% CI 4.2–5.4) and 5.3 (95% CI 4.2–5.5), respectively. Moreover, the AUHSROC in the subgroup of measurement within 6 h after commencing HFNC was comparable to that in the subgroup of measurement during 6–12 h. The stratified analyses also suggested that the ROC index was a reliable predictor of HFNC success in pneumonia patients with coronavirus disease 2019.

**Conclusions:**

In pneumonia patients with AHRF, the ROX index measured within 12 h after HFNC initiation is a good predictor of successful weaning from HFNC. The range of 4.2–5.4 may represent the optimal confidence interval for the prediction of HFNC outcome.

**Supplementary Information:**

The online version contains supplementary material available at 10.1186/s12890-022-01914-2.

## Background

Pneumonia complicated with acute hypoxemic respiratory failure (AHRF) is a severe disease in the intensive care unit (ICU). High-flow nasal cannula (HFNC) has been demonstrated as an effective respiratory support to prevent intubation in such population [1–4]. Through delivering a high flow of warmed humidified gas, HFNC generates a series of physiological effects on the respiratory function, including dead-space washout, generation of positive airway pressure, increase in end-expiratory lung volume, and reduction of work of breathing [5–8]. In this regard, HFNC can be implemented as an alternative to invasive mechanical ventilation (IMV) in some cases. However, one great concern on the application of HFNC should be highlighted, that is potentially delayed intubation, which was reported to be associated with prolonged duration of IMV and worse prognosis [9]. Hence, it is necessary to discriminate against those AHRF patients who will succeed with HFNC and those who will fail, as early as possible.

Recently, the respiratory rate-oxygenation (ROX) index, defined as the ratio of pulse oximetry (SpO_2_)/fraction of inspired oxygen (FiO_2_) to respiratory rate (RR), was proposed to predict the outcome of HFNC in pneumonia patients with AHRF [10, 11]. In recent years, more and more evidence suggests the predictive ability of the ROX index for the outcome of HFNC in pneumonia patients with AHRF [12–14]. However, the diagnostic accuracies and the optimal threshold values of the ROX index largely varied across these studies. Given that the predictive performance of the ROX index has not yet been systematically evaluated, we conducted this systematic meta-analysis to assess the diagnostic accuracy of the ROX index for the successful weaning from HFNC in pneumonia patients with AHRF. Furthermore, we also estimated the range of the optimal threshold value.

## Method

This systematic meta-analysis was conducted in accordance with the Preferred Reporting Items for a Systematic Review and Meta-analysis of Diagnostic Test Accuracy [15]. The study protocol was registered at the international prospective register of systematic reviews (PROSPERO, CRD42021274788) before study initiation.

### Data sources and search strategy

The PubMed, Embase, Web of Science, and Cochrane Library were systematically searched through August 24, 2021, by two independent authors (Zhou X and Liu J) in our review team to retrieve studies that evaluated the diagnostic accuracy of the ROX index for the success or failure of HFNC application in pneumonia patients with AHRF. We conducted a secondary search on February 12, 2022, to add the latest literatures. The search strategies are presented in Additional file 1. We also manually searched the bibliographies of relevant publications to further identify relevant literature. This meta-analysis had no date or language restriction.

### Criteria for inclusion and exclusion

To minimize the heterogeneities among the included studies, we established stringent eligibility criteria to screen candidate studies. The inclusion criteria included: (1) observational or randomized studies that enrolled pneumonia adults (age > 18 years) with AHRF who were initiated on HFNC after conventional oxygen therapy failure; (2) whether patients succeed in weaning from HFNC was considered as the reference index; (3) the ROX index was measured within 12 h after the HFNC onset and considered as the index test, and; (4) studies reported sufficient information to construct a 2 × 2 contingency table. Candidate studies were ineligible if they met one of the following criteria: (1) studies on patients who did not receive HFNC, patients who received noninvasive mechanical ventilation (NIV) before the HNFC onset, or patients who received HFNC after extubation; (2) studies did not report data on the sensitivity or specificity that was identified by the maximum of Youden index; (3) studies measured the ROX index after 12 h of HFNC onset; (4) studies with a sample size of less than 30, or; (5) conference abstracts without a full text.

### Definition

The ROX index was defined as the ratio of SpO_2_/FiO_2_ to RR, and the HFNC success was defined as the successful weaning from HFNC. The HFNC failure and the criteria for diagnosing AHRF and indicating HFNC application were defined by the authors in the included trials. According to the previous publications [13, 14], we predefined AHRF as the presence of a RR more than 25 breaths/min with SpO_2_ less than 92%, and/or the arterial oxygen partial pressure (PaO_2_) to FiO_2_ ratio less than 300 despite conventional oxygen therapy at 10 L/min. We also predefined HFNC failure as escalation to mechanical ventilation (non-invasive or invasive) or death.

### Study selection and data extraction

All searched records were initially checked for duplicates. After deduplication, two authors (Pan J and Xu Z) independently reviewed the title and abstract of the remaining records for eligibility. And then, they independently read the full text of candidate articles to determine whether they met the inclusion criteria or not. A third reviewer (Xu J) participated in the discussion to adjudicate any disagreements between the two reviewers. We recorded the reasons for precluding ineligible studies in detail in the Additional file 2: Table S1.

The same two independent authors (Pan J and Xu Z) pre-customized two extraction forms (the baseline characteristics form and the diagnostic accuracy form) to extract associated data from each included study. The baseline characteristics included the study characteristics and patient characteristics. The diagnostic accuracy form recorded the area under the receiver operating characteristic (ROC) curve (AUROC), the cutoff value of the ROX index, and the sensitivity and specificity. In studies that measured the ROX index at multiple time points within 12 h after the HFNC onset, we included one of the multiple measurements (with the largest value of sensitivity plus specificity) in the primary analysis. The same had been done in the subgroup analyses of measurements within 6 h and during 6–12 h after the HFNC onset. To construct a 2 × 2 contingency table, we computed the true positive, false positive, false negative, and true negative values according to the sensitivity, specificity, and sample size in each included study. In those studies that did not report such data, we returned to the original ROC curve to determine the optimal cutoff point, which represents the maximum of the sensitivity plus the specificity, and estimated corresponding sensitivity and specificity, otherwise we contacted the authors to inquire about the missing data of interest. Disagreements between the two reviewers were resolved by a joint review to reach a consensus.

### Quality assessment

Two authors (Zhou X and Liu J) in our review team independently evaluated the methodological quality of each included study using the Quality Assessment of Diagnostic Accuracy Studies (QUADAS)-2 tool [16]. Disagreements were resolved by consensus. The QUADAS-2 tool consists of two parts of assessment: the risk of bias and applicability concerns. The risk of bias assessment involves four domains: the patient selection, index test, reference standard, and flow and timing. Assessment of applicability concerns on the first three domains is also required.

### Statistical analysis

Before data synthesis, we plotted estimates of the paired observed sensitivities and specificities from each study on forest plot and ROC space to detect the variations in the diagnostic accuracy between studies [17]. The between-study variations were expected because the patient and study characteristics and the cutoff values of the ROX index largely varied across included studies. We adopted the hierarchical summary ROC (HSROC) model to fit an HSROC curve and used the random-effect bivariate model, which takes into account the possible correlation between sensitivity and specificity, to summary the pooled sensitivity, specificity, and diagnostic odds ratio (DOR) along with corresponding 95% confidence intervals (CI) [18]. Publication bias was assessed by using Deeks’ funnel plot asymmetry test [19]. Statistical analyses were conducted using Stata/SE 15.0 software with the MIDAS and METANDI modules (Stata-Corp, College Station, TX, USA). A two-tailed *P* < 0.05 indicated statistical significance.

Heterogeneity within studies was assessed by Cochran’s Q test and I^2^ statistics, and the threshold effect was evaluated graphically by visual inspection of the HSROC curve and statistically by calculating the Spearman correlation coefficient between the logit of sensitivity and the logit of 1-specificity [20]. Due to the expected threshold effect in this meta-analysis, we reported the area under the HSROC curve (AUHSROC) as the primary measure of diagnostic accuracy [18]. Theoretically, it is unreasonable to pool the sensitivity and specificity as measures of diagnostic accuracy because estimates for a certain notional unspecified average of different thresholds are clinically uninterpretable [17, 18]. To overcome this limitation, we estimated the optimal threshold value of the ROX index by observing the distribution, dispersion, central tendency, and extremum of the cut-off values as well as calculating the mean and median cut-off values. Meanwhile, we constructed a Bayesian nomogram to calculate the post-test probability to facilitate the interpretation of the findings.

Since the measurement time might affect the diagnostic accuracy of the ROX index, we conducted a subgroup analysis to observe the difference of diagnostic accuracy between measurement within 6 h and measurement during 6–12 h after HFNC initiation. Stratified analyses were also performed based on the type of pneumonia [coronavirus disease 2019 (COVID-19) or not] and the study design (prospective or retrospective). Given that several included studies had no explicit definition of AHRF, we conducted a sensitivity analysis to confirm the stability of the results by restricting the analysis to studies that met our criteria for diagnosing AHRF.

## Results

### Study selection

**Fig. 1:**
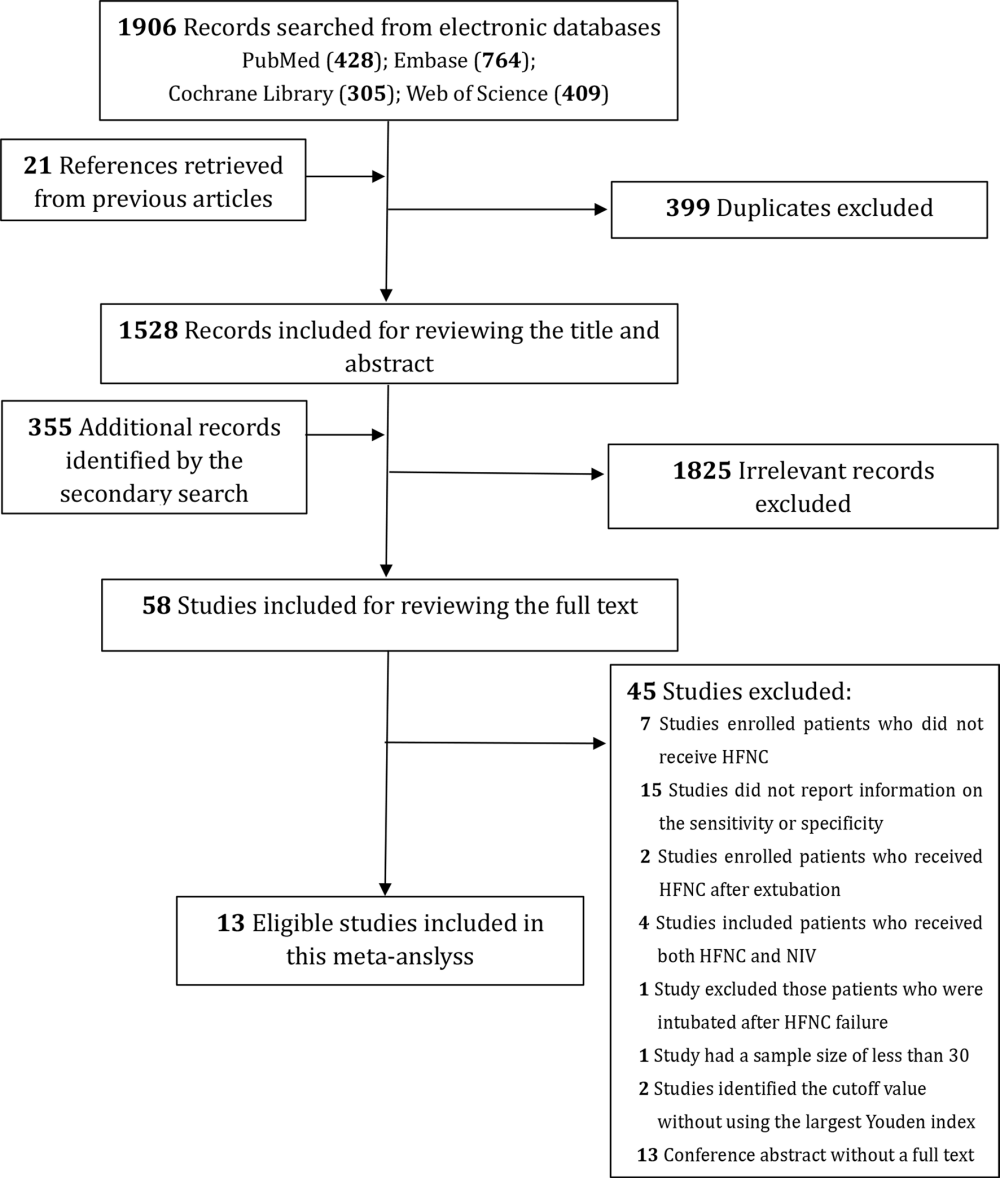
Flowchart of the study selection in this study. *HFNC* high-flow nasal cannula, *NIV* non-invasive ventilation

A total of 1906 citations were identified from electronic databases and additional 21 records were manually searched from previous publications. The secondary search identified 355 additional records. After deduplication and excluding irrelevant citations, we reviewed the full text of the remaining 58 records carefully. Finally, thirteen studies [10, 12, 13, 21–30] were deemed as eligible and included in this meta-analysis. The PRISMA flowchart of study inclusion is presented in Fig. 1.

### Baseline characteristics

Among the 13 included studies, six [10, 12, 13, 21, 29, 30] were prospectively designed and 7 [22–28] were retrospectively designed, and 10 [12, 21–28, 30] recruited AHRF patients with COVID-19-associated pneumonia. The sample size ranged from 30 to 324. Five studies [13, 21, 23, 24, 27] reported the PaO_2_/FiO_2_ ratio at baseline which ranged from 68 to 194 mmHg. The durations of HFNC application were largely different among the included studies, which ranged from 16.2 h to 3 days in the failure group and from 41.5 to 242 h in the success group. Nine studies [10, 12, 13, 21, 24, 25, 27, 29, 30] explicitly defined the criteria to diagnose AHRF or indicate HFNC application. Of note, the criteria for suggesting HFNC application in one study [12] did not fulfill the criteria to diagnose AHRF in our study. The remaining 4 studies [22, 23, 26, 28] did not report the definition of AHRF. Ten studies [12, 21–28, 30] reported the ROX index with available diagnostic accuracy that measured within 6 h after the HFNC onset, and seven studies [10, 13, 22, 23, 26, 29, 30] reported the ROX index with available diagnostic accuracy that measured during 6–12 h after the HFNC onset. Details on the baseline characteristics and the diagnostic accuracy are presented in Table 1 and Additional file 2: Tables S2 and S3.

**Table 1:** The baseline characteristics

Study no	Author/year	Design	Location	Subjects	Sample size	Age (years; mean or median)	PaO_2_/FiO_2_ ratio at baseline (mmHg; mean or median)	The ROX index at baseline (mean or median)	Duration of HFNC		Mortality (death/total)	
									HFNC success	HFNC failure	HFNC success	HFNC failure
1	Blez/2020	Prospective, single-centre	France	COVID-19 pneumonia patients treated with HFNC	30	64	NR	NR	5 (4–7) days	1 (0.9–2.5) day	NR	NR
2	Calligaro/2020	Prospective, multi-centre	South Africa	COVID-19 pneumonia patients with AHRF	293	52	68	NR	6 (3–9) days	2 (1–5) days	1/137	129/156
3	Chandel/2021	Retrospective, multi-centered	USA	COVID-19 pneumonia patients with AHRF	272	57	NR	NR	4 (2–7) days	2 (1–4) days	NR	49/108
4	Duan/2021	Retrospective, multi-centered	China	COVID-19 pneumonia patients with AHRF	66	67	194	9.0	242 (144–295) hours	39 (15–117) hours	0/37	14/29
5	Daniel/2021	Prospective, multi-centre	Colombia and Bolivia	Pneumonia patients with AHRF	106	59	NR	NR	NR	NR	7/27	8/79
6	Ferrer/2021	Prospective, single-centre	Spain	COVID-19 pneumonia patients with AHRF	85	65	NR	NR	3.29 ± 0.53	1.47 ± 0.21	2/38	17/47
7	Goh/2020	Prospective, single-centre	Singapore	Patients with AHRF (88% due to pneumonia)	99	64	93	4.0	41.5 (22.1–70.1) hours	16.2 (7.4–35.5) hours	11/54	27/45
8	Hu/2020	Retrospective, multi-centered	China	COVID-19 pneumonia patients with AHRF	105	64	116	NR	6.0 (3.5–8.5) days	3.0 (2.0–11.0) days	0/65	16/40
9	Panadero/2020	Retrospective, single-centre	Spain	COVID-19 pneumonia patients with AHRF	40	59	NR	3.8	6 (5–8) days	2 (1–4) days	0/19	9/21
10	Roca/2016	Prospective, multi-centre	Spain and France	Pneumonia patients with AHRF	157	52	NR	6.9	3 (2–6) days	1 (1–4) days	NR	NR
11	Vega/2022	Retrospective, multi-centered	Argentina and Italy	COVID-19 pneumonia patients with AHRF	120	NR	NR	NR	NR	NR	0/85	9/35
12	Xu/2020	Retrospective, multi-centered	China	COVID-19 pneumonia patients with AHRF	324	63	141	4.3	10 (7–15) days	3 (1–4) days	NR	NR
13	Zucman/2020	Retrospective, single-centre	France	COVID-19 pneumonia patients with AHRF	62	55	NR	NR	NR	NR	0/21	2/41

*COVID-19* coronavirus disease 2019, *AHRF* acute hypoxemic respiratory failure, *MV* mechanical ventilation, *HFNC* high-flow nasal cannula, *ROX* respiratory rate-oxygenation, *PaO*_*2*_ arterial oxygen partial pressure, *FiO*_*2*_ fraction of inspired oxygen, *NR* no record

### Methodological quality

Overall, none of the included studies was judged as having a high methodological quality. Three studies [13, 22, 26] were at high risk of bias in the patient selection because of the inappropriate exclusion of patients who were transitioned to non-invasive mechanical ventilation (NIV) after HFNC failure, which might result in a reduced rate of HFNC failure. As the severe shortage of medical devices might affect the decision to intubate, the study by Duan et al. [23] was judged as having a high risk of bias in the reference standard domain. Notably, two studies [12, 13] had a high applicability concern on the patient selection. One [12] enrolled subjects who did not meet the definition criteria of AHRF in this meta-analysis, and a small part of patients in the other one [13] had no diagnosis of pneumonia. The methodological quality of each included study is summarized in Table 2.

**Table 2:**
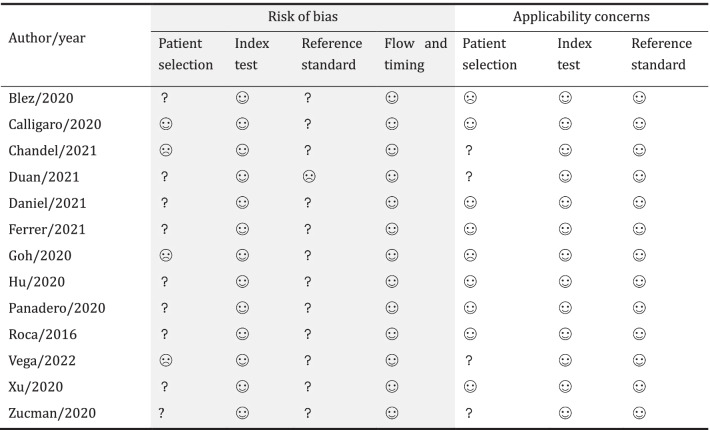
Assessment of methodological quality of each study

low risk;  high risk; ? unclear risk

### Primary analysis of the diagnostic accuracy of the ROX index

**Fig. 2:**
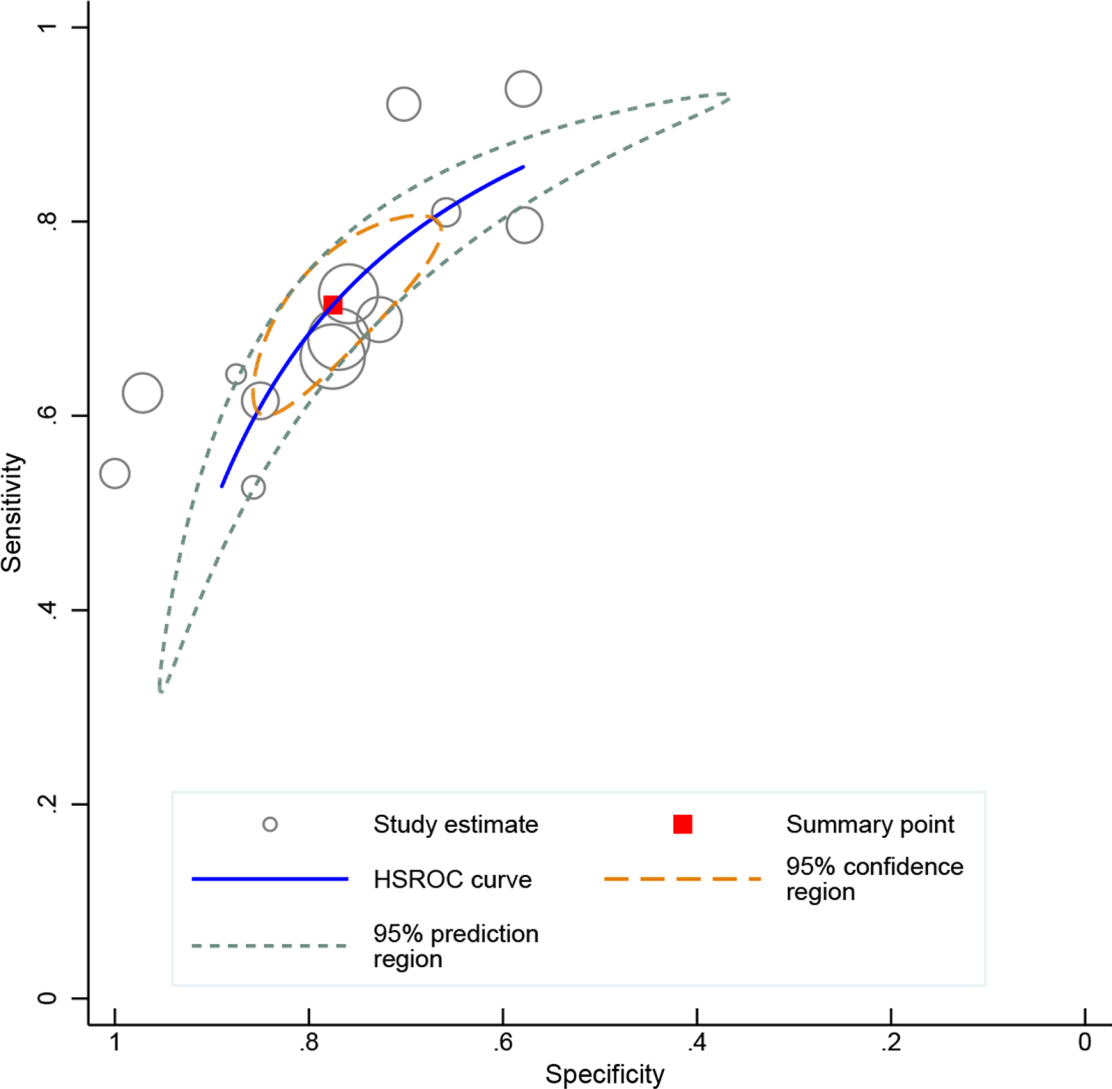
HSROC curve of the ROX index for predicting the successful weaning from HFNC. The area under the hierarchical summary receiver operating curve was 0.81 (95% CI 0.77–0.84). The size of the circles indicates the weight of each individual study. *HSROC* hierarchical summary receiver operating characteristic, *ROX* respiratory rate-oxygenation, *HFNC* high-flow nasal cannula

**Fig. 3:**
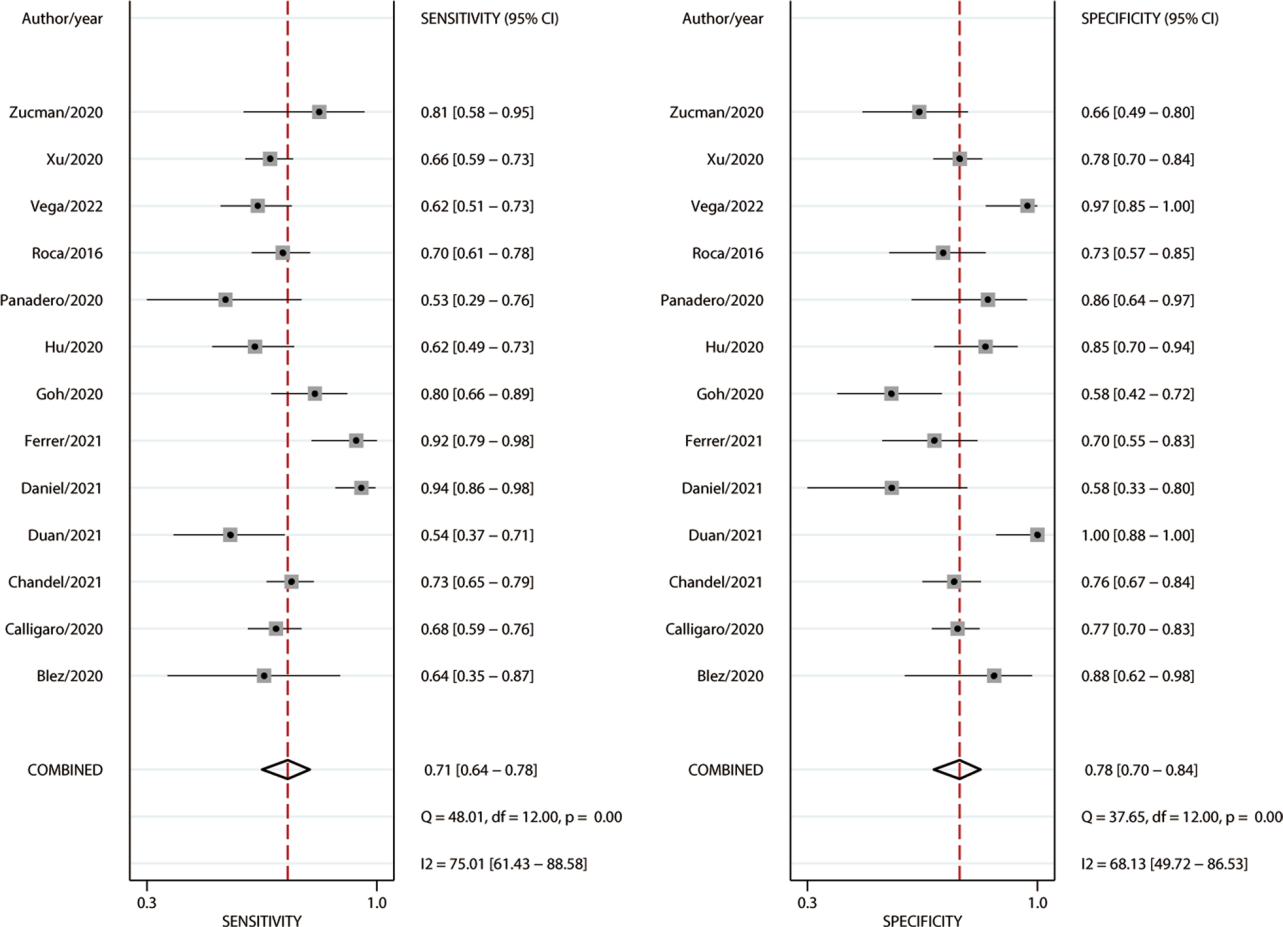
Forest plot of sensitivity and specificity of the ROX index for predicting the successful weaning from HFNC. *ROX* respiratory rate-oxygenation, *HFNC* high-flow nasal cannula

**Fig. 4:**
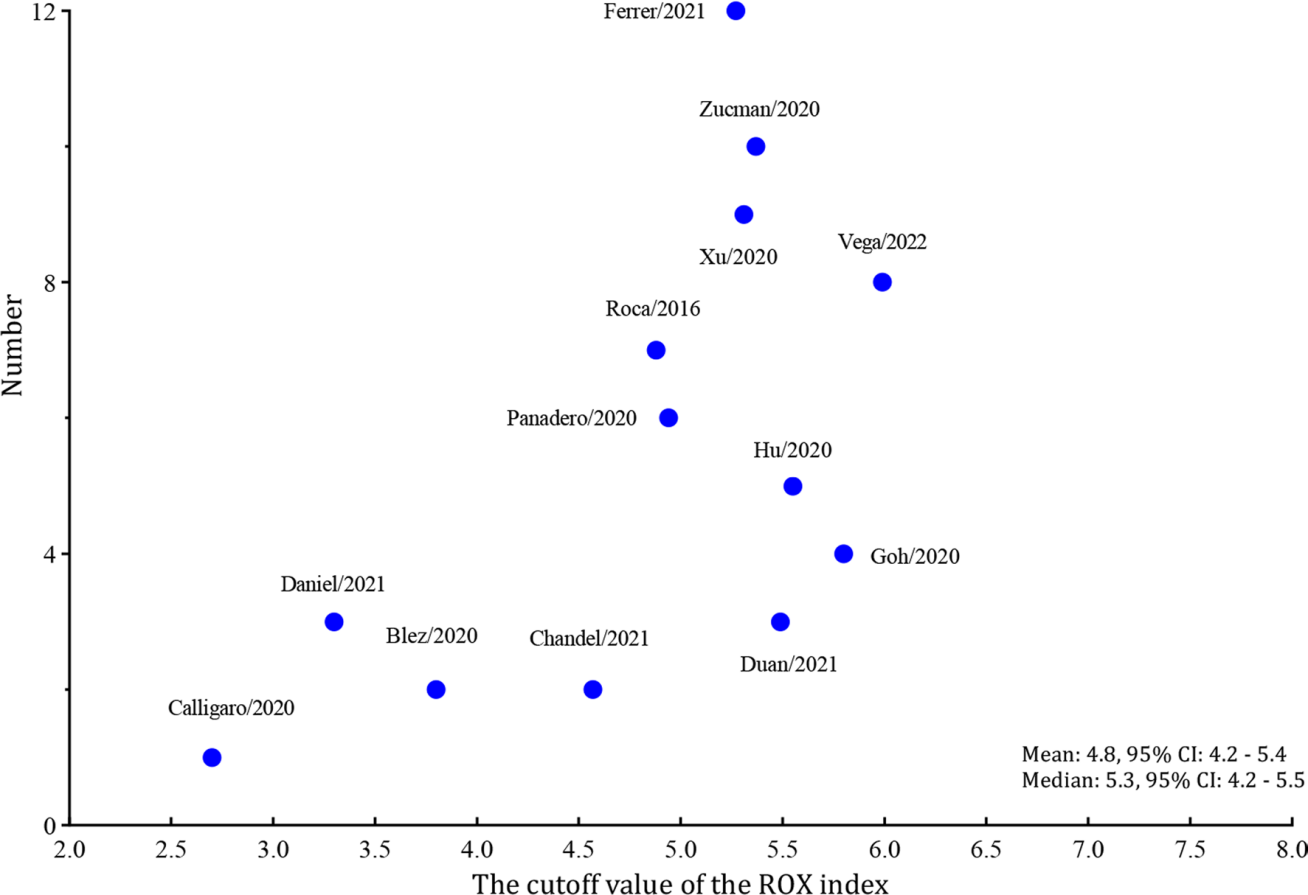
Scatter plot of the cut-off values of the ROX index. The cutoff values were nearly conically symmetrically distributed and most data were centered between 4.5 and 6.0. *ROX* respiratory rate-oxygenation

Among the 1751 patients enrolled in the 13 studies, 1003 (57.3%) successfully weaned from HFNC. Significant between-study heterogeneities were found with a Cochran Q statistic of 40.849 (*P* < 0.001) and an overall I^*2*^ of 95%. All the heterogeneities likely resulted from the threshold effect that was confirmed by visual inspection of the HSROC curve (Fig. 2) and the Spearman correlation coefficient (ρ = − 1.0). Overall, the ROX index measured within 12 h after the HFNC onset exhibits good performance for predicting the successful weaning from HFNC in pneumonia patients with AHRF, with an AUHSROC of 0.81 (95% CI 0.77–0.84), a DOR of 8.3 (95% CI 6.4–10.8), a pooled sensitivity of 0.71 (95% CI 0.64− 0.78), and a pooled specificity of 0.78 (95% CI 0.70–0.84) (Table 3 and Fig. 3). All included studies reported the cutoff value of the ROX index, which varied from 2.7 to 5.99. As shown in the scatter plot (Fig. 4), the cutoff values of the ROX index were nearly conically symmetrically distributed and most data were centered between 4.5 and 6.0. The mean and median cutoff values were 4.8 (95% CI 4.2–5.4) and 5.3 (95% CI 4.2–5.5), respectively.

**Table 3:** Effect estimates of the ROX index for predicting the successful weaning from HFNC

Variables	Grouping	No. of studies	No. of subjects		AUHSROC	Sensitivity (%)	Specificity (%)	Diagnostic odds ratio	Estimated optimal cutoff value	
			HFNC success	HFNC failure					Mean value (95% CI)	Median value (95% CI)
*Primary analysis*	All studies	13	1003	748	0.81 (0.77, 0.84)	0.71 (0.64, 0.78)	0.78 (0.70, 0.84)	8.3 (6.4, 10.8)	4.8 (4.2, 5.4)	5.3 (4.2, 5.5)
*Subgroup analyses*										
Measurement time point	Within 6 h after HFNC onset	10	757	640	0.80 (0.76, 0.83)	0.66 (0.56, 0.75)	0.79 (0.72, 0.84)	7.3 (5.6, 9.4)	5.0 (4.2, 5.8)	5.3 (4.0, 5.8)
	During 6–12 h after HFNC onset	7	570	327	0.84 (0.81, 0.87)	0.77 (0.64, 0.85)	0.80 (0.61, 0.91)	11.5 (6.4, 20.7)	5.0 (4.2, 5.9)	5.3 (3.9, 5.9)
Pneumonia type	COVID-19	10	757	640	0.79 (0.75, 0.82)	0.67 (0.61, 0.73)	0.82 (0.74, 0.88)	8.7 (6.4, 11.8)	4.9 (4.2, 5.6)	5.3 (4.2, 5.5)
	Non-COVID-19	3	246	108	NE	NE	NE	NE	NE	NE
Study design	Prospective	6	435	327	0.79 (0.75, 0.82)	080 (0.68, 0.88)	0.70 (0.62, 0.77)	8.6 (5.4, 13.5)	4.3 (3.0, 5.6)	4.3 (2.8, 5.7)
	Retrospective	7	568	421	0.75 (0.71, 0.78)	0.65 (0.59, 0.71)	0.86 (0.74, 0.93)	8.4 (5.8, 12.1)	5.3 (4.9, 5.7)	5.4 (4.8, 5.8)
*Sensitivity analysis*	Excluding studies without explicit AHRF definition	8	682	519	0.80 (0.76, 0.83)	0.75 (0.64, 0.84)	0.73 (0.66, 0.80)	7.6 (5.7, 10.0)	4.7 (3.8, 5.6)	5.1 (3.2, 5.6)

*HFNC* high-flow nasal cannula, *COVID-19* coronavirus disease 2019, *AHRF* acute hypoxemic respiratory failure, *NE* not estimable, *CI* confidential interval

A Bayes nomogram (Additional file 2: Fig. S1) was constructed to facilitate the interpretation of the findings. Based on the estimated rate of HFNC success in this meta-analysis, if an average-risk population has an assumed pretest probability of HFNC success of 60%, the probability of HFNC success will increase to 83% when the test result is positive and decrease to 36% when the test result is negative. The Deeks’ funnel plot asymmetry test suggested no significant publication bias (*P* = 0.12) (Additional file 2: Fig. S2).

### Subgroup analyses and sensitivity analysis

In the subgroup analyses, the diagnostic accuracy of the ROX index that measured within 6 h after the HFNC onset was comparable to that of measurement during 6–12 h after the HFNC onset (Additional file 2: Figs. S3–S6). The stratified analyses also suggested that the ROC index was a reliable predictor of HFNC success in patients with COVID-19-associated pneumonia, with an AUHSROC of 0.79 (95% CI 0.75–0.82) (Additional file 2: Figs. S7 and S8). The mean and median cutoff values were 4.9 (95% CI 4.2–5.6) and 5.3 (95% CI 4.2–5.5), respectively (Table 3). The study design might have no impact on the diagnostic accuracy of the ROX index. After excluding five studies [12, 22, 23, 26, 28] that did not meet our AHRF definition criteria, the AUHSROC in the sensitivity analysis was similar to that in the primary analysis (Table 3), indicating the robustness of the results.

## Discussion

This systematic meta-analysis demonstrated that the ROX index performed well in predicting the successful separation from HFNC in pneumonia patients with AHRF, irrespective of the measurement within 6 h or during 6–12 h after the HFNC onset. The outcome of the HFNC application may be not predicted reliably when the measured ROX index is between 4.2 and 5.4, which may represent the optimal confidence interval for the prediction of HFNC outcome. In addition, the ROX index is also a good predictor of HFNC outcome in patients with AHRF related to COVID-19 pneumonia. These findings suggest the necessity to dynamically monitor the ROX index during the early period of HFNC application.

Recently, marked variability in the timing of intubation for patients with AHRF was observed among different ICUs [31], and late intubation was associated with a worse prognosis in AHRF patients treated with HFNC [9, 32]. Thus, it is always a point of special interest for physicians to explore when to intubate patients who will fail on HFNC. Based on the significant association between some respiratory variables (such as SpO_2_, PaO_2_/FiO_2_, and RR) and HFNC failure [33, 34], Roca et al. [10] proposed a new variable, termed as ROX index (i.e., the (SpO_2_/FiO_2_)/RR ratio), and validated its ability to predict HFNC success in pneumonia patients with AHRF [14]. Afterward, numerous studies confirmed the predictive value for the outcomes of HFNC in the same population. Through systematically searching the literature and pooling the currently available data, this meta-analysis concluded that the ROX index exhibits good performance for predicting the successful weaning from HFNC with high specificity (true negative), indicating a high value for identifying those patients who will suffer from HFNC failure.

In this study, we chose the ROX index that measured within 12 h, but not after 12 h, of HFNC initiation as the index test because the duration of HFNC in the HFNC failure group varied from 16.2 h to 3 days (Table 1). Thus, measurement after 12 h of HFNC initiation may be inappropriate because a considerable part of patients were intubated before measuring the ROX index. One may raise a question that when is the optimal measurement time for the ROX index to predict the outcome of HFNC. Several studies [10, 13, 14, 24, 26] found a significant improvement in the ROX index overtime after the use of HFNC, and the changes of the ROX index over time in the HFNC success group were obviously greater than that in the HFNC failure group. Thus, it may be reasonable to assume that the diagnostic accuracy of the ROX index will be also improved over time within 12 h after HFNC initiation. However, the subgroup analyses in this meta-analysis suggested a comparable diagnostic accuracy between the measurement within 6 h and the measurement during 6–12 h after HFNC application. In reality, it is not always true that the ROX index will be increased over time after the use of HFNC. In recently published studies [12, 22, 23], the ROX index did not change significantly after the use of HFNC in both the success and failure groups. The potential explanation for this phenomenon may be that the subjects included in these studies [12, 22, 23] had better oxygenation. For instance, the PaO_2_/FiO_2_ ratio at baseline in the study by Duan et al. [23] was higher than that in the studies by Goh et al. [13] and Hu et al. [24] (Table 1). It seems to be theoretically reasonable that the ROX index in patients with relatively better oxygenation before HFNC initiation maybe not well-responsive to the use of HFNC. In this consideration, we speculate that whether the diagnostic accuracy of the ROX index can be improved over time depends on the baseline oxygenation, and this hypothesis should be verified in future researches.

Because the ROX index consists of three commonly used respiratory variables that can be easily obtained and repeatedly monitored without requiring complex monitoring devices, it thus has become a routine monitoring parameter in patients treated with HFNC. In a realistic clinical decision-making scenario, using a single cutoff value seemly cannot meet the demand to predict the HFNC outcome. For instance, if the measured ROX index is slightly higher or lower than the cutoff value, it is difficult to determine which population will succeed or fail on HFNC. To overcome this limitation, we applied the ‘confidence interval’ approach to avoid the binary constraint of a “black-or-white” decision of the ROC curve and fit the reality of clinical or screening practice [35]. After observing the distribution, dispersion, and central tendency of the cut-off values, we estimated the optimal cutoff value and its corresponding confidence interval. The estimated mean cutoff value was 4.8, which was similar to the cutoff value in the study by Roca et al. [10], and its 95% CI range (4.2–5.4) was narrow and similar to that of the median value (4.2–5.5), indicating a robust confidence interval for the prediction of HFNC outcome. Therefore, a decision-making algorithm can be established: (1) if the measured ROX index is greater than 5.4, the patient is expected to have a high chance of success; (2) if it is less than 4.2, the patient is at high risk of HFNC failure and should be considered to require escalation of respiratory support; (3) if the measured ROX index is between 4.2 and 5.4, the HFNC outcome cannot be predicted reliably. In this case, a repeated measurement is suggested.

Consistent with the results of the meta-analysis by Prakash et al. [36], the subgroup analysis in the current study also revealed the discriminating power of the ROX index for predicting the HFNC outcome in COVID-19 patients. However, this current study has two main advantages over the previous one [36]. First, we conducted a comprehensive literature search to avoid missing literature, and 10 studies regarding COVID-19 patients were included. As we know, one study [23] was missed in their meta-analysis. Incomplete data syntheses might reduce the credibility of their evidence to some extent. Second, directly pooling sensitivities and specificities are unreasonable in the absence of a specified average of different thresholds because of a possible misleading interpretation of the clinical significance of the ROX index [17, 18]. Thus, we initially estimated the optimal cutoff value and corresponding 95% CI and used the AUHSROC as the primary measure of diagnostic accuracy. However, these procedures are lacking in the previous study [36]. Despite these strengths, our study still has several limitations. Firstly, some interesting subgroup analyses were not performed because of the limited number of related studies. For instance, the settings (ICU or not) and the baseline oxygenation (such as the PaO_2_/FiO_2_ ratio) may be associated with the diagnostic accuracy of the ROX index. Secondly, none of the included studies had a high methodological quality, and the varied patient and study characteristics partially contributed to the substantial heterogeneities among the included studies. The methodological shortcoming might intrinsically lead to a potential bias in our results, and the significant heterogeneities might represent a challenge to the reliability of our evidence. Thirdly, our study has a main clinical restriction that the current findings are only applicable to pneumonia patients with AHRF in whom the ROX index was measured within 12 h after the HFNC onset. The diagnostic value of the ROX index is unclear for those patients with others etiologies related to AHRF.

## Conclusion

In pneumonia patients with AHRF, the ROX index, measured within 6 h or during 6–12 h after HFNC initiation, exhibits good performance for predicting the successful weaning from HFNC, and the confidence interval of the ROX index for the prediction of HFNC outcome may be reliable in the range of 4.2–5.4. Given the low methodological quality of the included studies, more studies with high methodological quality are warranted to validate the applicability of the ROX index in the future.

## Supplementary Information

**Additional file 1**. Detailed search strategy in each database.

**Additional file 2**. Supplementary tables and figures.

## Data Availability

All data generated or analyzed during this study are included in this published article (and its Additional file 1 and Additional file 2).

## Abbreviations

AHRF: Acute hypoxemic respiratory failure
ICU: Intensive care unit
HFNC: High-flow nasal cannula
IMV: Invasive mechanical ventilation
ROX: Respiratory rate-oxygenation
SpO_2_: Pulse oximetry
FiO_2_: Fraction of inspired oxygen
RR: Respiratory rate
PaO_2_: Arterial oxygen partial pressure
ROC: Receiver operating characteristic
AUROC: Area under the ROC curve
HSROC: Hierarchical summary ROC
DOR: Diagnostic odds ratio
CI: Confidence intervals
AUHSROC: Area under the HSROC curve
COVID-19: Coronavirus disease 2019
NIV: Non-invasive mechanical ventilation
