# Supplementary material for: The ROX index as a predictor of high-flow nasal cannula outcome in pneumonia patients with acute hypoxemic respiratory failure: a systematic review and meta-analysis
Source: BMC Pulm Med. 2022 Apr 1;22:121. doi: 10.1186/s12890-022-01914-2 (PMC8972647; doi:10.1186/s12890-022-01914-2)
Supplement: Supplementary file 1 — Additional file 1. Detailed search strategy in each database. [file 12890_2022_1914_MOESM1_ESM.docx]

**Detailed search strategies in each database**

The four electronic databases were initially searched on August 24, 2021, and updated on February 12, 2022.

**PubMed: 428 records**

1. ((((((((((((((((((Bronchopneumonia) OR (Healthcare-Associated Pneumonia)) OR (Pneumonia, Ventilator-Associated)) OR (Pleuropneumonia)) OR (Pneumonia, Aspiration)) OR (Pneumonia, Lipid)) OR (Pneumonia, Bacterial)) OR (Chlamydial Pneumonia)) OR (Pneumonia of Calves, Enzootic)) OR (Pneumonia of Swine, Mycoplasmal)) OR (Pneumonia, Mycoplasma)) OR (Pneumonia, Pneumococcal)) OR (Pneumonia, Rickettsial)) OR (Pneumonia, Staphylococcal)) OR (Pneumonia, Necrotizing)) OR (Pneumonia, Pneumocystis)) OR (Pneumonia, Viral)) OR (COVID-19)) OR ("Pneumonia"[Mesh]) (337246 records)
2. ((((Cannula[MeSH Terms]) OR Nasal Cannula) OR Cannula, Nasal) OR Nasal Cannulae) OR Cannulae, Nasal (17374 records)
3. (high-flow) OR high flow (181413 records)
4. (#2 AND #3) (2111 records)
5. ((HFNC) OR HHFNC) OR HHFN (772 records)
6. ((((((oxygen inhalation therapy[MeSH Terms]) OR Inhalation Therapy, Oxygen) OR Inhalation Therapies, Oxygen) OR Oxygen Inhalation Therapies) OR Therapies, Oxygen Inhalation) OR Therapy, Oxygen Inhalation) OR oxygen therapy (127366 records)
7. #3 AND #6 (3833 records)
8. #4 OR #5 OR #7 (4866 records)
9. #1 AND #9 Filters: Humans (428 records)

**Embase: 764 records**

1. ('cannula'/exp OR 'cannula' OR 'nasal cannula'/exp OR 'nasal cannula' OR 'cannula, nasal' OR 'nasal cannulae' OR 'cannulae, nasal') AND ('high-flow' OR 'high flow') (4147 records)
2. ('oxygen inhalation therapy'/exp OR 'oxygen inhalation therapy' OR 'inhalation therapy, oxygen' OR 'inhalation therapies, oxygen' OR 'oxygen inhalation therapies' OR 'therapies, oxygen inhalation' OR 'therapy, oxygen inhalation' OR 'oxygen therapy'/exp OR 'oxygen therapy') AND ('high-flow' OR 'high flow') (2532 records)
3. 'hfnc' OR 'hhfnc' OR 'hhfn' (1524 records)
4. #1 OR #2 OR #3 (5131 records)
5. pneumonia OR bronchopneumonia OR ('healthcare associated' AND pneumonia) OR (pneumonia, AND 'ventilator associated') OR pleuropneumonia OR (pneumonia, AND aspiration) OR (pneumonia, AND lipid) OR (pneumonia, AND bacterial) OR (chlamydial AND pneumonia) OR (pneumonia AND of AND calves, AND enzootic) OR (pneumonia AND of AND swine, AND mycoplasmal) OR (pneumonia, AND mycoplasma) OR (pneumonia, AND pneumococcal) OR (pneumonia, AND rickettsial) OR (pneumonia, AND staphylococcal) OR (pneumonia, AND necrotizing) OR (pneumonia, AND pneumocystis) OR (pneumonia, AND viral) OR 'coronavirus disease 2019' (501666 records)
6. #4 AND #5 AND 'article'/it (764 records)

**Cochrane Central Register of Controlled Trials: 305 records**

1. MeSH descriptor: [Cannula] explode all trees (125 records)
2. (Nasal Cannula) OR (Cannula*, Nasal) OR (Nasal Cannula*) (1830 records)
3. #1 OR #2 (1850 records)
4. (high-flow) OR (high flow) (17831 records)
5. #3 AND #4 (1187 records)
6. (HFNC) OR (HHFNC) OR (HHFN) (562 records)
7. MeSH descriptor: [Oxygen Inhalation Therapy] explode all trees (1638 records)
8. (Inhalation Therapy, Oxygen) OR (Inhalation Therapies, Oxygen) OR (Oxygen Inhalation Therapies) OR (Therapies, Oxygen Inhalation) OR ((Therapy, Oxygen Inhalation) OR (oxygen therapy)) (21166 records)
9. #7 OR #8 (21243 records)
10. #4 AND #9 (2607 records)
11. #5 OR #6 OR #10 (3147 records)
12. MeSH descriptor: [Pneumonia] explode all trees (4402 records)
13. (Bronchopneumonia) OR (Healthcare-Associated Pneumonia) OR (Pneumonia, Ventilator-Associated) OR (Pleuropneumonia) OR (Pneumonia, Aspiration) OR (Pneumonia, Lipid) OR (Pneumonia, Bacterial) OR (Chlamydial Pneumonia) OR (Pneumonia of Calves, Enzootic) OR (Pneumonia of Swine, Mycoplasmal) OR (Pneumonia, Mycoplasma) OR (Pneumonia, Pneumococcal) OR (Pneumonia, Rickettsial) OR (Pneumonia, Staphylococcal) OR (Pneumonia, Necrotizing) OR (Pneumonia, Pneumocystis) OR (Pneumonia, Viral) OR (COVID-19) OR (Pneumonia) (23816 records)
14. #12 OR #13 (23816 records)
15. #11 AND #14, Limits: Trials (305 records)

**Web of Science: 409 records**

1. TS=(Nasal Cannula) OR TS=(Cannula, Nasal) OR TS=(Nasal Cannulae) OR TS=(Cannulae, Nasal) (3331 records)
2. TS=(high-flow) OR TS=(high flow) (764385 records)
3. #1 AND #2 (2199 records)
4. TS=(HFNC) OR TS=(HFNC) OR TS=(HHFN) (709 records)
5. TS=(oxygen inhalation therapy) OR TS=(Inhalation Therapy, Oxygen) OR TS=(Inhalation Therapies, Oxygen) OR TS=(Oxygen Inhalation Therapies) OR TS=(Therapies, Oxygen Inhalation) OR TS=(Therapy, Oxygen Inhalation) OR TS=(oxygen therapy) (63629 records)
6. #2 AND #5 (3446 records)
7. #3 OR #4 OR #6 (4546 records)
8. TS=(Pneumonia) OR TS=(COVID-19) OR TS=(Bronchopneumonia) OR TS=(Healthcare-Associated Pneumonia) OR TS=(Pneumonia, Ventilator-Associated) OR TS=(Pleuropneumonia) OR TS=(Pneumonia, Aspiration) OR TS=(Pneumonia, Lipid) OR TS=(Pneumonia, Bacterial) OR TS=(Chlamydial Pneumonia) (292286 records)
9. TS=(Pneumonia of Calves, Enzootic) OR TS=(Pneumonia of Swine, Mycoplasmal) OR TS=(Pneumonia, Mycoplasma) OR TS=(Pneumonia, Pneumococcal) OR TS=(Pneumonia, Rickettsial) OR TS=(Pneumonia, Staphylococcal) OR TS=(Pneumonia, Necrotizing) OR TS=(Pneumonia, Pneumocystis) OR TS=(Pneumonia, Viral)
10. #8 OR #9 (292286 records)
11. #7 AND #10, Publication type: article (409 records)
