# Supplementary material for: The ROX index as a predictor of high-flow nasal cannula outcome in pneumonia patients with acute hypoxemic respiratory failure: a systematic review and meta-analysis
Source: BMC Pulm Med. 2022 Apr 1;22:121. doi: 10.1186/s12890-022-01914-2 (PMC8972647; doi:10.1186/s12890-022-01914-2)
Supplement: Supplementary file 2 — Additional file 2. Supplementary tables and figures. [file 12890_2022_1914_MOESM2_ESM.docx]

**The ROX index as a predictor of high-flow nasal cannula outcome in pneumonia patients with acute hypoxemic respiratory failure – a systematic review and meta-analysis**

**ADDITIONAL FILE 2.**

**Table of contents**

[Table S1. Ineligible studies and the reasons for exclusion 2](#_Toc97628381)

[Table S2. Detailed diagnostic accuracy of the ROX index in each included study 6](#_Toc97628382)

[Table S3. Definition of HFNC failure in each included study 8](#_Toc97628383)

[Figure S1. Bayes nomogram of the ROX index for the prediction of HFNC success 10](#_Toc97628384)

[Figure S2. Assessment of publication bias 11](#_Toc97628385)

[Figure S3. Forest plot of sensitivity and specificity of the ROX index measured within 6 hours after HFNC initiation for the prediction of HFNC success 12](#_Toc97628386)

[Figure S4. HSROC of the ROX index measured within 6 hours after HFNC initiation for the prediction of HFNC success 13](#_Toc97628387)

[Figure S5. Forest plot of sensitivity and specificity of the ROX index measured during 6-12 hours after HFNC initiation for the prediction of HFNC success 14](#_Toc97628388)

[Figure S6. HSROC of the ROX index measured during 6-12 hours after HFNC initiation for the prediction of HFNC success 15](#_Toc97628389)

[Figure S7. Forest plot of sensitivity and specificity of the ROX index for the prediction of HFNC success in COVID-19 patients 16](#_Toc97628390)

[Figure S8. HSROC of the ROX index for the prediction of HFNC success in COVID-19 patients 17](#_Toc97628391)

# Table S1. Ineligible studies and the reasons for exclusion

| **Reasons for exclusion** | **Studies** |
| --- | --- |
| Studies included subjects who did not receive HFNC (7 studies) | Gianstefani/2021 [1]; Mukhtar/2021 [2]; Suliman/2021 [3]; Zaboli/2021 [4]; Cattazzo/2022 [5]; Gianstefani/2021 [6]; Prower/2021 [7] |
| Studies did not report information on the sensitivity or specificity to construct a 2×2 contingency table (15 studies) | Bonnet/2021 [8]; Kim/2020 [9]; Celejewska-Wójcik/2021 [10]; Mellado-Artigas/2021 [11]; Xia/2020 [12]; Zemach/2019 [13]; Varipapa/2021 [14]; Alshahrani/2021 [15]; Chavarria/2021 [16]; Kerai/2022 [17]; Molini/2022 [18]; Myers/2022 [19]; Panadero/2020 [20]; Patel/2021 [21]; Takeshita/2022 [22] |
| Studies included subjects who received HFNC after extubation (2 studies) | Helviz/2020 [23]; Liu/2021 [24]; |
| Studies included subjects who received both HFNC and NIV (4 studies) | Liu/2021 [25]; Lemiale/2021 [26]; Colaianni-Alfonso/2021 [27];  Lun/2022 [28] |
| The study excluded those patients who were intubated after HFNC failure (no the first attempt to wean from HFNC) | Rodriguez/2019 [29] |
| The study had a sample size of less than 30 | Artacho Ruiz/2021 [30]; |
| Studies identified the cutoff value without using the largest Youden index method (2 studies) | Roca/2019 [31]; Valencia/2021 [32] |
| Conference abstract without a full text (13 studies) | Kumar/2021 [33]; Carroll/2021 [34]; Cable/2020 [35]; Cysneiros/2020 [36]; D'Espiney/2020 [37]; Reyes/2019 [38]; Nguyen/2021 [39]; Patel/2021 [40]; Bruna/2021 [41]; Butler/2021 [42]; Ghabbara/2021 [43]; Gupta/2021 [44]; Patil/2021 [45] |

**Reference**

1. Gianstefani A, Farina G, Salvatore V, Alvau F, Artesiani ML, Bonfatti S, et al. Role of ROX index in the first assessment of COVID-19 patients in the emergency department. Intern Emerg Med. 2021. doi: 10.1007/s11739-021-02675-2.
2. Mukhtar A, Rady A, Hasanin A, Lotfy A, El Adawy A, Hussein A, et al. Admission SpO2 and ROX index predict outcome in patients with COVID-19. Am J Emerg Med. 2021; 50:106-110.
3. Suliman LA, Abdelgawad TT, Farrag NS, Abdelwahab HW. Validity of ROX index in prediction of risk of intubation in patients with COVID-19 pneumonia. Adv Respir Med. 2021;89(1):1-7.
4. Zaboli A, Ausserhofer D, Pfeifer N, Sibilio S, Tezza G, Ciccariello L, et al. The ROX index can be a useful tool for the triage evaluation of COVID-19 patients with dyspnoea. J Adv Nurs. 2021;77(8):3361-3369.
5. Cattazzo F, Inglese F, Dalbeni A, Piano S, Pengo MF, Montagnana M, et al; COVID Internal Medicine Team. Performance of non-invasive respiratory function indices in predicting clinical outcomes in patients hospitalized for COVID-19 pneumonia in medical and sub-intensive wards: a retrospective cohort study. Intern Emerg Med. 2022:1–10. doi: 10.1007/s11739-021-02922-6.
6. Gianstefani A, Farina G, Salvatore V, Alvau F, Artesiani ML, Bonfatti S, et al. Role of ROX index in the first assessment of COVID-19 patients in the emergency department. Intern Emerg Med. 2021;16(7):1959-1965.
7. Prower E, Grant D, Bisquera A, Breen CP, Camporota L, Gavrilovski M, et al. The ROX index has greater predictive validity than NEWS2 for deterioration in Covid-19. EClinicalMedicine. 2021;35:100828.
8. Bonnet N, Martin O, Boubaya M, Levy V, Ebstein N, Karoubi P, et al. High flow nasal oxygen therapy to avoid invasive mechanical ventilation in SARS-CoV-2 pneumonia: a retrospective study. Ann Intensive Care. 2021;11(1):37.
9. Kim BK, Kim S, Kim CY, Cha J, Lee YS, Ko Y, et al. Factors Associated with Failure of High-Flow Nasal Cannula. Respir Care. 2020;65(9):1276-1284.
10. Celejewska-Wójcik N, Polok K, Górka K, Stachura T, Kania A, Nastałek P, et al. High-flow nasal oxygen therapy in the treatment of acute respiratory failure in severe COVID-19 pneumonia: a prospective observational study. Pol Arch Intern Med. 2021;131(7-8):658-665.
11. Mellado-Artigas R, Mujica LE, Ruiz ML, Ferreyro BL, Angriman F, Arruti E, et al; COVID-19 Spanish ICU Network. Predictors of failure with high-flow nasal oxygen therapy in COVID-19 patients with acute respiratory failure: a multicenter observational study. J Intensive Care. 2021;9(1):23.
12. Xia J, Zhang Y, Ni L, Chen L, Zhou C, Gao C, et al. High-Flow Nasal Oxygen in Coronavirus Disease 2019 Patients With Acute Hypoxemic Respiratory Failure: A Multicenter, Retrospective Cohort Study. Crit Care Med. 2020;48(11):e1079-e1086.
13. Zemach S, Helviz Y, Shitrit M, Friedman R, Levin PD. The Use of High-Flow Nasal Cannula Oxygen Outside the ICU. Respir Care. 2019;64(11):1333-1342.
14. Varipapa RJ Jr, DiGiacomo E, Jamieson DB, Desale S, Sonti R. Fluid Balance Predicts Need for Intubation in Subjects With Respiratory Failure Initiated on High-Flow Nasal Cannula. Respir Care. 2021;66(4):566-572.
15. Alshahrani MS, Alshaqaq HM, Alhumaid J, Binammar AA, AlSalem KH, Alghamdi A, et al. High-Flow Nasal Cannula Treatment in Patients with COVID-19 Acute Hypoxemic Respiratory Failure: A Prospective Cohort Study. Saudi J Med Med Sci. 2021;9(3):215-222.
16. Chavarria AP, Lezama ES, Navarro MG, Vazquez RRV, Bello HH, Gascon JL,et al. High-flow nasal cannula therapy for hypoxemic respiratory failure in patients with COVID-19. Ther Adv Infect Dis. 2021;8: 20499361211042959. doi: 10.1177/20499361211042959.
17. Kerai S, Singh R, Saxena KN, Desai SD, Bhalotra AR. A Retrospective Study on Experience of High-flow Nasal Cannula Oxygen in Critically Ill COVID-19 Adult Patients Admitted to Intensive Care Unit. Indian J Crit Care Med. 2022;26(1):62-66.
18. Molini WJ, Gonzalez RM, Villalba LM, Ruiz EF, Diocares LA, Anticura DB, et al. High-flow nasal therapy in severe respiratory failure due to SARS-CoV-2. Medicina (B Aires). 2022;82(1):47-54. Spanish.
19. Myers LC, Mark D, Ley B, Guarnieri M, Hofmeister M, Paulson S, et al. Validation of Respiratory Rate-Oxygenation Index in Patients With COVID-19-Related Respiratory Failure. Crit Care Med. 2022. doi: 10.1097/CCM.0000000000005474.
20. Panadero C, Abad-Fernández A, Rio-Ramirez MT, Acosta Gutierrez CM, Calderon-Alcala M, Lopez-Riolobos C, et al. High-flow nasal cannula for Acute Respiratory Distress Syndrome (ARDS) due to COVID-19. Multidiscip Respir Med. 2020;15(1):693.
21. Patel M, Chowdhury J, Mills N, Marron R, Gangemi A, Dorey-Stein Z, et al. Utility of the ROX Index in Predicting Intubation for Patients With COVID-19-Related Hypoxemic Respiratory Failure Receiving High-Flow Nasal Therapy: Retrospective Cohort Study. JMIRx Med. 2021;2(3):e29062.
22. Takeshita Y, Terada J, Hirasawa Y, Kinoshita T, Tajima H, Koshikawa K, et al. High-flow nasal cannula oxygen therapy in hypoxic patients with COVID-19 pneumonia: A retrospective cohort study confirming the utility of respiratory rate index. Respir Investig. 2022;60(1):146-153.
23. Helviz Y, Hajaj T, Burger A, Levin PD, Einav S. A Retrospective Study of Short-term versus Long-term Use of High Flow Nasal Cannula after Extubation in the Intensive Care Unit. Isr Med Assoc J. 2020;22(3):173-177.
24. Liu T, Zhao Q, Du B. Effects of high-flow oxygen therapy on patients with hypoxemia after extubation and predictors of reintubation: a retrospective study based on the MIMIC-IV database. BMC Pulm Med. 2021;21(1):160.
25. Liu L, Xie J, Wu W, Chen H, Li S, He H, et al. A simple nomogram for predicting failure of non-invasive respiratory strategies in adults with COVID-19: a retrospective multicentre study. Lancet Digit Health. 2021;3(3):e166-e174.
26. Lemiale V, Dumas G, Demoule A, Pène F, Kouatchet A, Bisbal M, et al; Groupe de Recherche en Reanimation Respiratoire du patient d’Onco-Hématologie (GRRR-OH). Performance of the ROX index to predict intubation in immunocompromised patients receiving high-flow nasal cannula for acute respiratory failure. Ann Intensive Care. 2021;11(1):17.
27. Colaianni-Alfonso N, Montiel G, Castro-Sayat M, Siroti C, Vega ML, Toledo A, et al. Combined non-invasive respiratory support therapies to treat SARS-CoV-2 patients: A prospective Observational Study. Respir Care. 2021: respcare.09162.
28. Lun CT, Leung CK, Shum HP, So SO. Predictive factors for high-flow nasal cannula failure in acute hypoxemic respiratory failure in an intensive care unit. Lung India. 2022;39(1):5-11.
29. Rodriguez M, Thille AW, Boissier F, Veinstein A, Chatellier D, Robert R, et al. Predictors of successful separation from high-flow nasal oxygen therapy in patients with acute respiratory failure: a retrospective monocenter study. Ann Intensive Care. 2019;9(1):101.
30. Artacho Ruiz R, Artacho Jurado B, Caballero Güeto F, Cano Yuste A, Durbán García I, García Delgado F, et al. Predictors of success of high-flow nasal cannula in the treatment of acute hypoxemic respiratory failure. Med Intensiva (Engl Ed). 2021;45(2):80-87.
31. Roca O, Caralt B, Messika J, Samper M, Sztrymf B, Hernández G, et al. An Index Combining Respiratory Rate and Oxygenation to Predict Outcome of Nasal High-Flow Therapy. Am J Respir Crit Care Med. 2019;199(11):1368-1376.
32. Valencia CF, Lucero OD, Castro OC, Sanko AA, Olejua PA. Comparison of ROX and HACOR scales to predict high-flow nasal cannula failure in patients with SARS-CoV-2 pneumonia. Sci Rep. 2021;11(1):22559.
33. Kumar R., Khan M.S., Banerjee S., Prakash J., Bhattacharya P.K. Impact of high-flow nasal oxygen therapy in COVID-19 critically ill patients with acute hypoxemic respiratory failure: A prospective observational study. Indian J Crit Care Med. 2021; 25 (SUPPL 1): S27.
34. Carroll R., Balasubramaniam R., Sawyer M., Tran K. A novel non-invasive index of oxygenation and prediction of outcomes for patients on high-flow nasal cannula. Respirology. 2021;26 (SUPPL 2): 186.
35. Cable C., Kashiouris M., Gross A., Wiese B. Utility of the respiratory rate-oxygenation (ROX) index in predicting respiratory failure requiring mechanical ventilation in acute care medicine. Chest 2020; 158:4(SUPPL): A576.
36. Cysneiros A., Organista D., MacHado D., Rodrigues D., Lopes F., Antunes C., et al. Using the Rox index for Nasal High Flow therapy in patients with interstitial lung disease and severe acute respiratory failure. Eur Respir J. 2020; 56(SUPPL 64).
37. D'Espiney Madeira Barbara R.M., Vincent J., Kelly J., MacAvei V., Martin-Lazaro J. Validity of ROX Index for Nasal High Flow Therapy in Critical Care. Eur Respir J. 2020; 56(SUPPL 64).
38. Reyes L.F., Arango A.J., Barros-Toro D., Cardona-Marín S., Carvajal-Canizales K., Marcela P., et al. The respiratory rate-oxygenation (ROX) index predict failure of postextubation high-flow nasal cannula (HFNC) therapy in ICU patients. Intensive Care Med Experimental. 2019; 7(SUPPL 3).
39. Nguyen S, Gupta M, Manek G, Datta D. Admission Respiratory Rate-Oxygenation (ROX) Index and Outcome in COVID-19 Pneumonia with Acute Respiratory Failure. Am J Respir Crit Care Med. 2021; 203: A3829.
40. Patel M, Chowdhury JM, Mills N, Marron RM, Gangemi AJ, Dorey-Stein ZL, et al. ROX Index Predicts Intubation in Patients with COVID-19 Pneumonia and Moderate to Severe Hypoxemic Respiratory Failure Receiving High Flow Nasal Therapy. Am J Respir Crit Care Med. 2021; 203: A2624.
41. Bruna M., Hidalgo G., Castaneda S., Galvez M., Bravo D., Benitez R., et al. Sensitivity or specificity for a predictor? Diaphragmatic ultrasound predictors of failure with high-flow nasal cannula as ventilatory therapy in patients with respiratory failure secondary to SARS-CoV-2 pneumonia. Intensive Care Medicine Experimental. 2021; 9: SUPPL 1.
42. Butler K., Patel J., Hodson J., Weblin J. Assessing the Predictive Accuracy of the ROX index with ITU patients admitted for High-Flow Nasal Oxygen with confirmed COVID-19. Intensive Care Medicine Experimental. 2021; 9:SUPPL 1.
43. Ghabbara R., Ayed S., Jamoussi A., Rachdi E., Jarraya F., Benkhelil J. Utility of high-flow nasal oxygen therapy for severe COVID-19 pneumonia. Intensive Care Medicine Experimental. 2021; 9:SUPPL 1.
44. Gupta M., Nguyen S., Manek G., Datta D. PREDICTING OUTCOMES IN COVID-19 PNEUMONIA WITH ACUTE RESPIRATORY FAILURE USING THE RESPIRATORY RATE-OXYGENATION INDEX. Chest. 2021; 160:4 Supplement (A1122-A1123).
45. Patil S. ROX INDEX A PROMISING NONINVASIVE INDEX. Chest. 2021; 160:4 Supplement (A1140).

## Table S2. Detailed diagnostic accuracy of the ROX index in each included study

| Study No. | Author/year | Measurement time point included for analysis | AUROC with 95% CI | Cutoff value | Sensitivity (%) | Specificity (%) | True positive | False positive | False negative | True negative |
| --- | --- | --- | --- | --- | --- | --- | --- | --- | --- | --- |
| 1 | Blez/2020 | 0.5 h after HFNC initiation | 0.78 (0.58 - 0.95)* | 3.8 | 64.2 | 87.5 | 9 | 2 | 5 | 14 |
| 2 | Calligaro/2020 | 6 h after HFNC initiation | 0.75 | 2.7 | 68 | 77 | 93 | 36 | 44 | 120 |
| 3 | Chandel/2021 | 6 h after HFNC initiation | 0.72 (0.65 - 0.79)* | NR | 65 | 75 | 107 | 27 | 57 | 81 |
|  |  | 12 h after HFNC initiation | 0.78 (0.72 - 0.84) | 4.57 | 72.4 | 75.9 | 119 | 26 | 45 | 82 |
| 4 | Duan/2021 | 4 h after HFNC initiation | 0.73 (0.57 - 0.86) | 6.25 | 63 | 86 | 23 | 4 | 14 | 25 |
|  |  | 12 h after HFNC initiation | 0.75 (0.60 - 0.86) | 5.49 | 55 | 100 | 20 | 0 | 17 | 29 |
| 5 | Daniel/2021 | 12 h after HFNC initiation | 0.76 (0.62 - 0.9) | 3.3 | 95 | 58 | 74 | 8 | 5 | 11 |
| 6 | Ferrer/2021 | 6 h after HFNC initiation | 0.83 (0.71- 0.95) | 5.25 | 96 | 64 | 36 | 17 | 2 | 30 |
|  |  | 12 h after HFNC initiation | 0.88 (0.77 - 0.99) | 5.27 | 93 | 71 | 35 | 14 | 3 | 33 |
| 7 | Goh/2020 | 10 h after HFNC initiation | 0.723 (0.605 - 0.840) | 5.8 | 78.26 | 58.62 | 43 | 19 | 11 | 26 |
| 8 | Hu/2020 | 6 h after HFNC initiation | 0.798 (0.703 - 0.893) | 5.55 | 61.1 | 84.6% | 40 | 6 | 25 | 34 |
| 9 | Panadero/2020 | 2-6 h after HFNC initiation | 0.712 | 4.94 | 52.6 | 85.7 | 10 | 3 | 9 | 18 |
| 10 | Roca/2016 | 12 h after HFNC initiation | 0.74 (0.64 – 0.84) | 4.88 | 70.1 | 72.4 | 79 | 12 | 34 | 32 |
| 11 | Vega/2022 | 6 h after HFNC initiation | 0.64 (0.51 - 0.78) | 5.8 | 41 | 90 | 35 | 3 | 50 | 32 |
|  |  | 12 h after HFNC initiation | 0.78 (0.67 - 0.89) | 5.99 | 62 | 96 | 53 | 1 | 32 | 34 |
| 12 | Xu/2020 | 4 h after HFNC initiation | 0.779 (0.73–0.83) | 5.31 | 66.1 | 77.6 | 117 | 33 | 60 | 114 |
| 13 | Zucman/2020 | Within 4 h after HFNC initiation | 0.75 (0.6 - 0.9) | 5.37 | 81 | 66 | 17 | 14 | 4 | 27 |

* Studies just reported the AUROC value of the ROX index without the sensitivity and specificity. Thus, we return to the original receiver operating characteristic curve in their article to identify the optimal cutoff point and estimate its corresponding sensitivity and specificity.

HFNC high-flow nasal cannula; AUROC area under the receiver operating characteristic curve; CI confidential interval; NR no record.

# Table S3. Definition of HFNC failure in each included study

| Study No. | Author / year | Definition of HFNC failure | The criteria for diagnosing AHRF or indicating HFNC application | Time points to measure the ROX index |
| --- | --- | --- | --- | --- |
| 1 | Blez/2020 | The need for invasive MV within 7 days of HFNC onset | An oxygen requirement above 6 L/min with a respiratory rate > 20/min, and/or a rapid worsening of the clinical status despite conventional oxygen therapy (+4L/min O_2_ and/or +10/min RR over the last 6 hours) | 0.5, 2, and 6 hours after HFNC initiation |
| 2 | Calligaro/2020 | The need for intubation or death whilst on HFNC | A RR ≥ 30 breaths per minute with oxygen saturations ≤ 92% despite oxygen at 15 L/min via reservoir bag, and/or PaO_2_/FiO_2_ ratio < 150 | 6 hours after HFNC initiation |
| 3 | Chandel/2021 | The need for intubation and MV | No record | 2, 6, and 12 hours after HFNC initiation |
| 4 | Duan/2021 | The requirement of NIV or intubation until discharge or death in the hospital | No record | 1, 2, 4, 8, 12, and 24 hours after HFNC initiation |
| 5 | Daniel/2021 | The need for intubation and invasive mechanical ventilation | Signs of respiratory distress or refractory hypoxemia in a low-flow system, with PaO_2_ < 60 mm Hg and PaO_2_/FiO_2_ < 200 | 2, 6, 12, 18, and 24 hours after HFNC initiation |
| 6 | Ferrer/2021 | Respiratory support upgraded to mechanical ventilation (non-invasive or invasive) or death | At least one of the three following criteria in patients on conventional oxygen therapy: severe dyspnea with signs of more labored breathing and use of accessory respiratory muscles, RR ≥ 30 breaths per minute, and PaO_2_/FiO_2_ < 200 mm Hg | 1, 6, 12, and 24 hours after HFNC initiation |
| 7 | Goh/2020 | The need for intubation and MV until death or discharge from hospital | A respiratory rate > 25 breaths/min and a PaO_2_/FiO_2_ ratio of < 300 mmHg on an oxygen device delivering 10 ≥ L/min, in the absence of chronic respiratory failure | 1, 2, 4, 6, 8, 10, 12, 18, 24 and 48 hours after HFNC initiation |
| 8 | Hu/2020 | The need for NIV or invasive MV and/or death while on HFNC support | Patients with SpO_2_ ≤ 92% and/or RR ≥ 25 times/min under nasal tube oxygen inhalation 10L/min or mask oxygen supply | 2, 6, 12, and 24 hours after HFNC initiation |
| 9 | Panadero/2020 | The need for intubation and MV within 30 days | A PaO_2_/FiO_2_ <200mmHg or SpO_2_/FiO_2_ ratio <240, and treated with high-flow oxygen therapy through nasal cannula | 2 – 6 hours after HFNC initiation |
| 10 | Roca/2016 | The need for invasive MV until death or hospital discharge | A SpO_2_ ≤ 92% and an RR ≥ 25 breaths per min while receiving standard oxygen administered through a face mask at 10 L or more | 2, 6, 12, 18, and 24 hours after HFNC initiation |
| 11 | Vega/2022 | Escalation to invasive MV or death | No record | 2, 6, 12, and 24 hours after HFNC initiation |
| 12 | Xu/2020 | The need for non-invasive or invasive MV within 7 days of HFNC initiation | A SpO_2_ of ≤ 90% or a RR >30 breaths per min despite conventional oxygen therapy with oxygen flow higher than 10 L/min or showed persistent signs of respiratory distress | Within 4 hours after HFNC initiation |
| 13 | Zucman/2020 | The need for intubation or death within 4 days of HFNC onset | No record | Within 4 hours after HFNC initiation |

HFNC high-flow nasal cannula; MV mechanical ventilation; NIV non-invasive ventilation; AHRF acute hypoxemic respiratory failure; RR respiratory rate.

# Figure S1. Bayes nomogram of the ROX index for the prediction of HFNC success


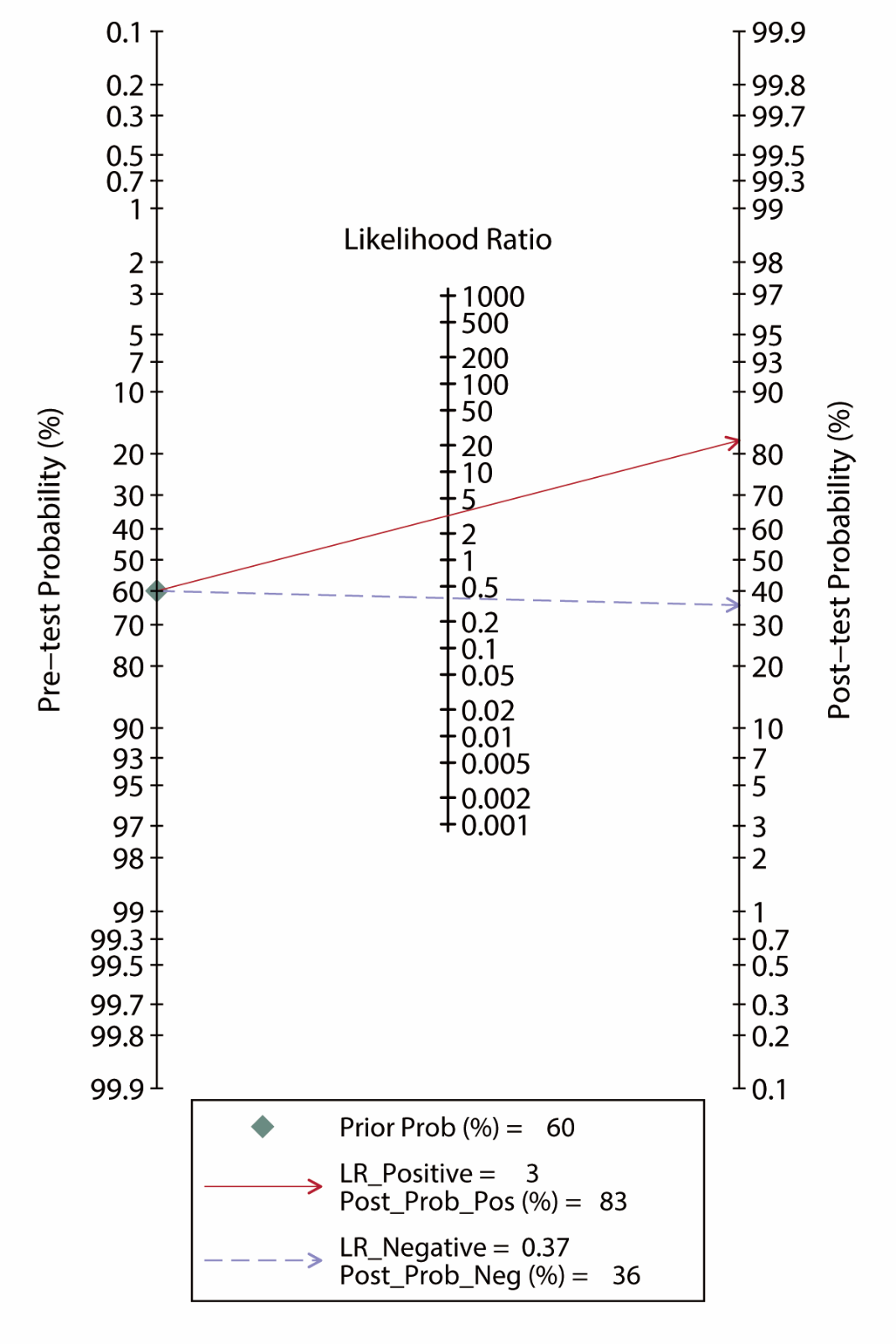


# Figure S2. Assessment of publication bias


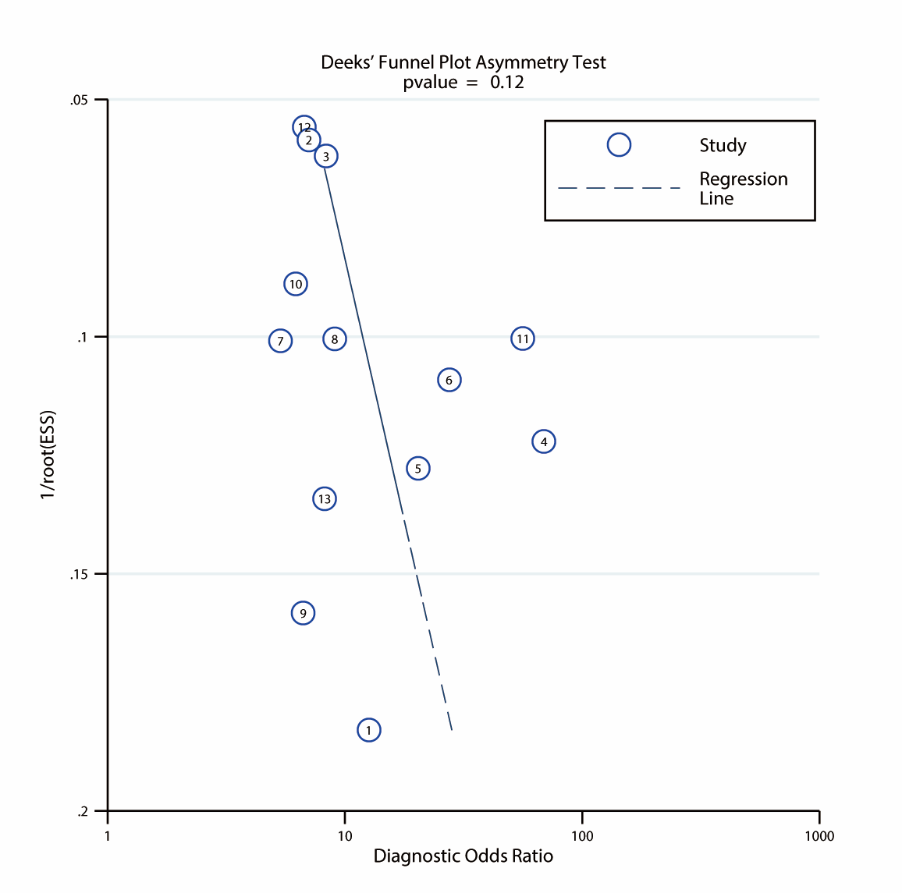


# Figure S3. Forest plot of sensitivity and specificity of the ROX index measured within 6 hours after HFNC initiation for the prediction of HFNC success


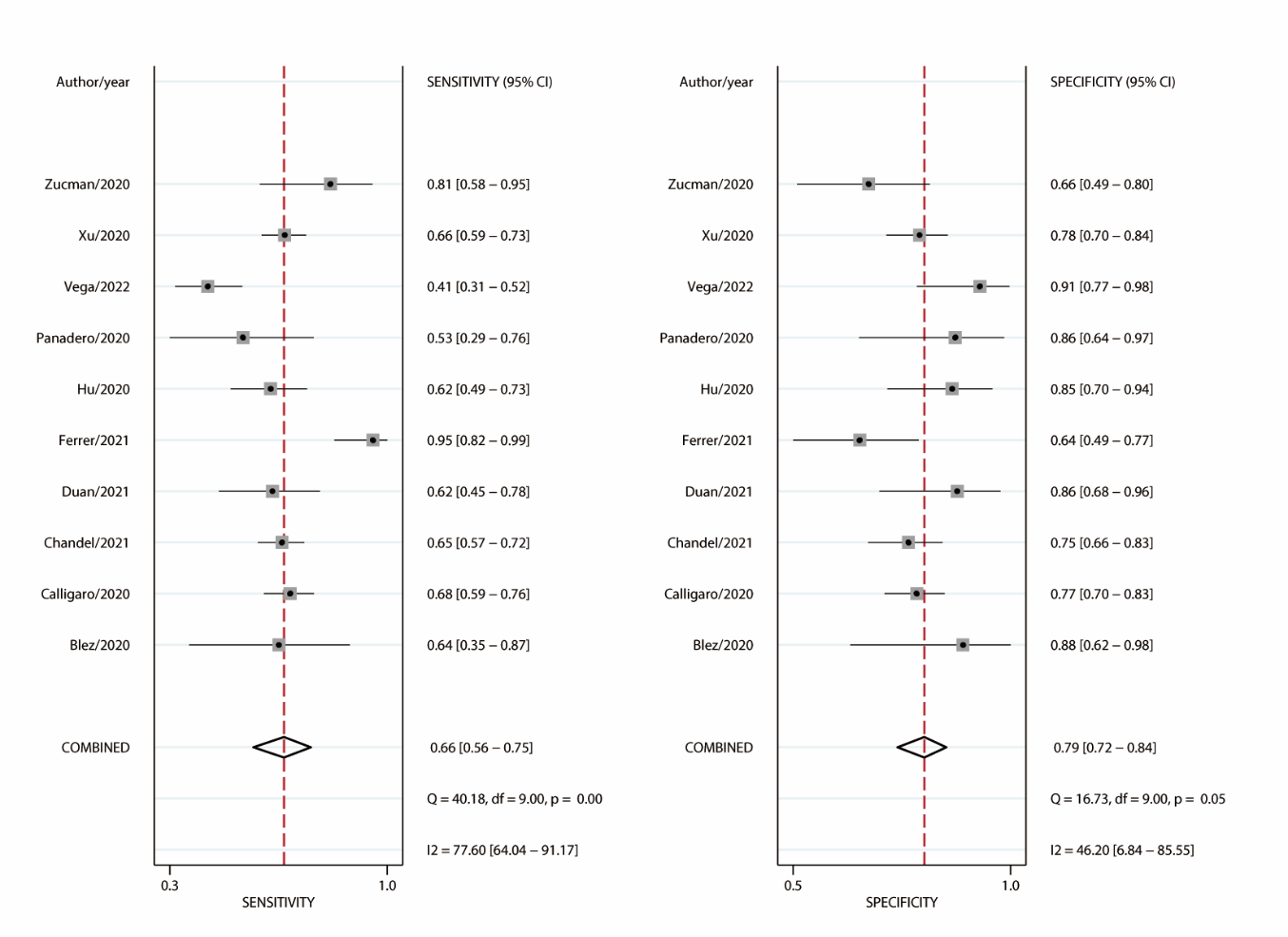


# Figure S4. HSROC of the ROX index measured within 6 hours after HFNC initiation for the prediction of HFNC success


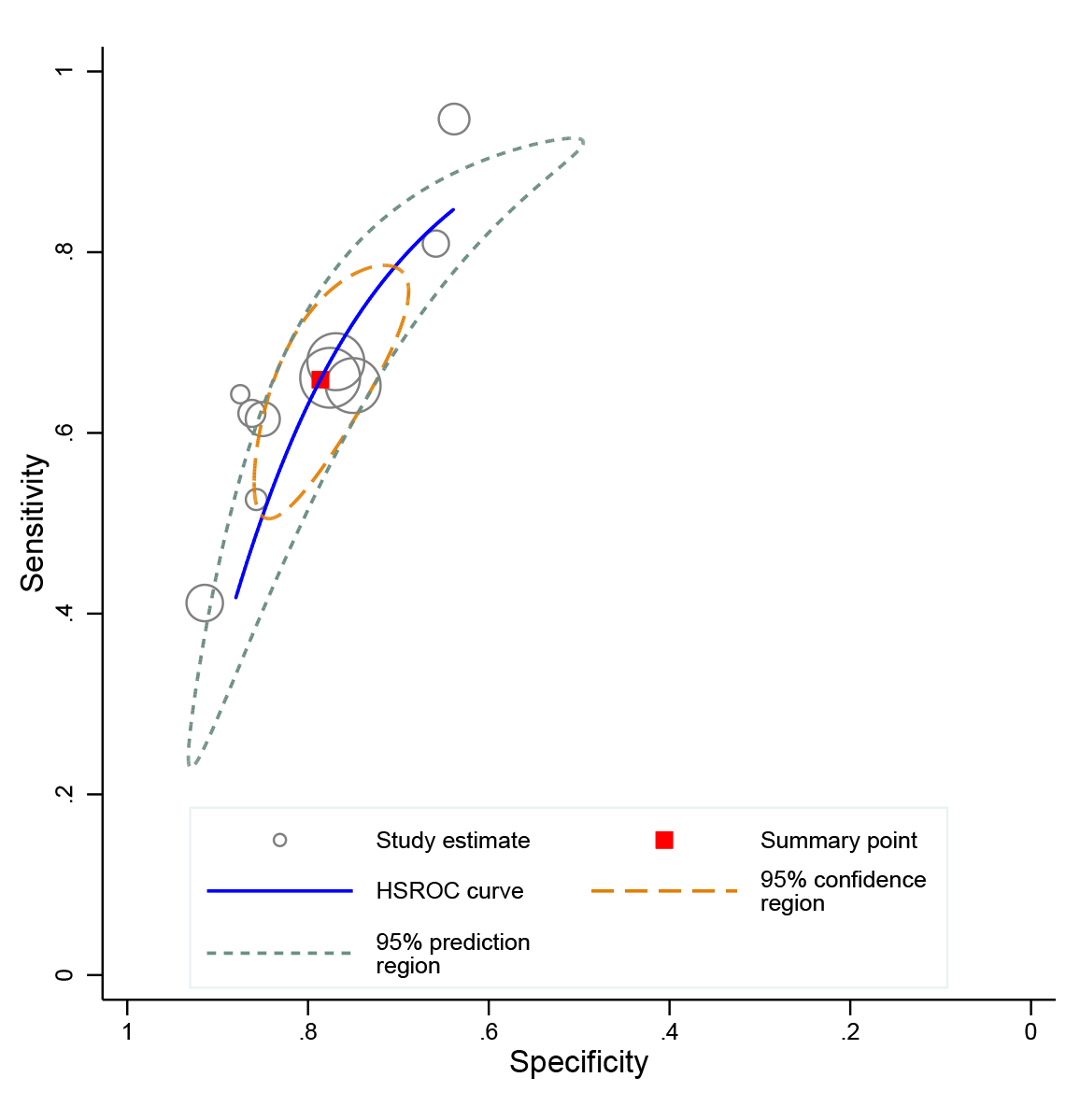


The area under the hierarchical summary receiver operating curve was 0.80 (95% CI: 0.76 to 0.83). The size of the circles indicates the weight of each individual study.

# Figure S5. Forest plot of sensitivity and specificity of the ROX index measured during 6-12 hours after HFNC initiation for the prediction of HFNC success


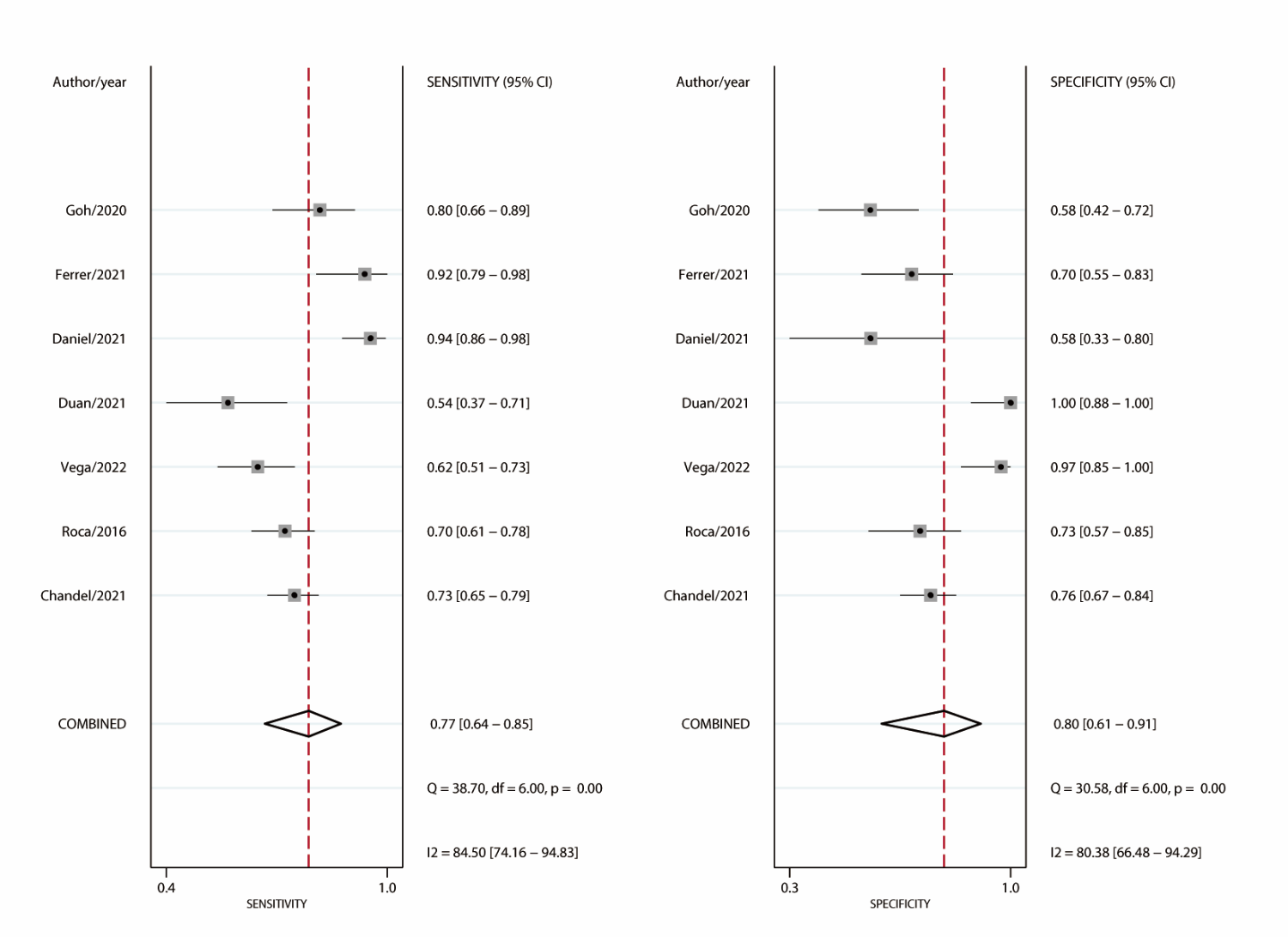


# Figure S6. HSROC of the ROX index measured during 6-12 hours after HFNC initiation for the prediction of HFNC success


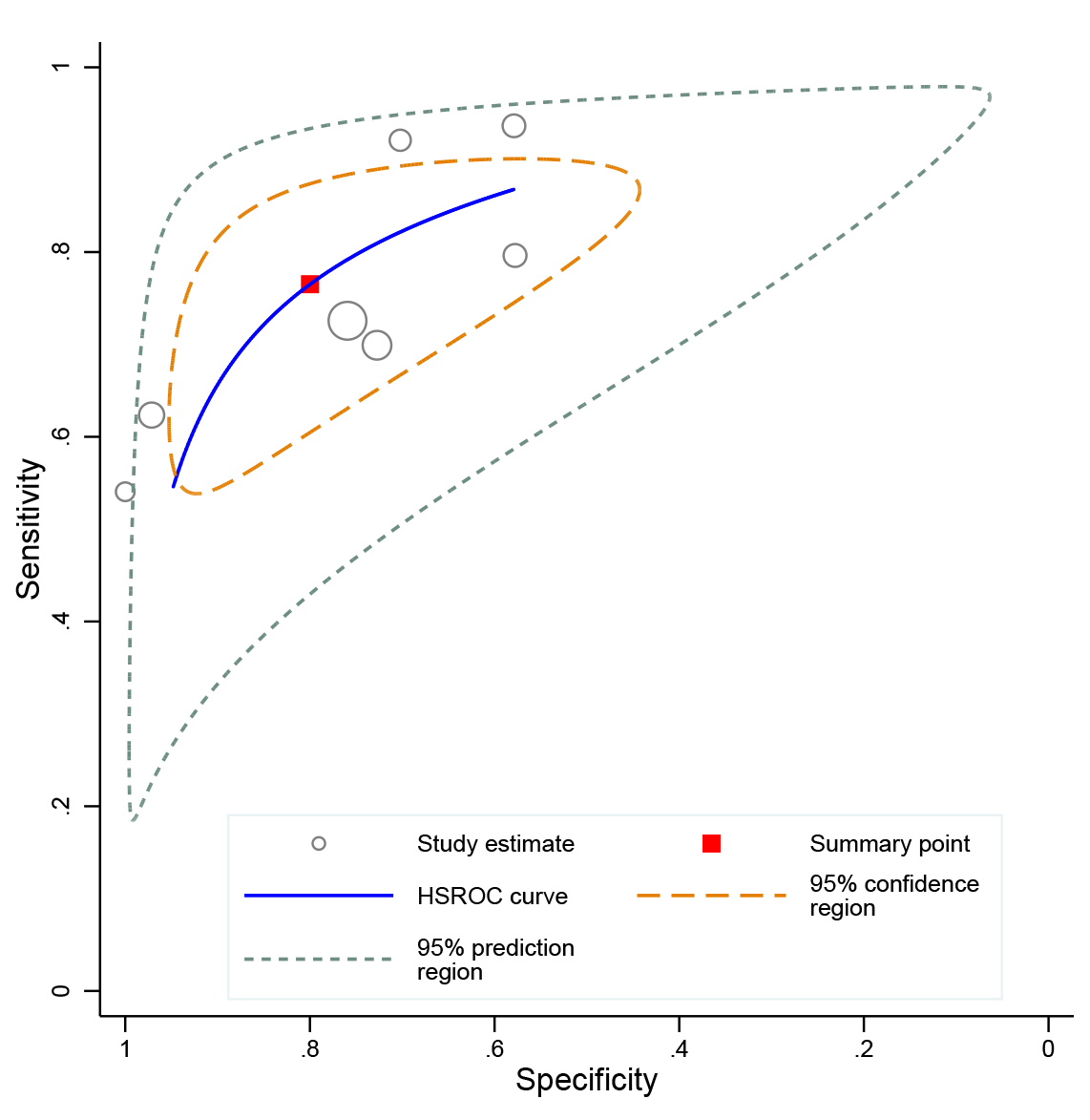


The area under the hierarchical summary receiver operating curve was 0.84 (95% CI: 0.81 to 0.87). The size of the circles indicates the weight of each individual study.

# Figure S7. Forest plot of sensitivity and specificity of the ROX index for the prediction of HFNC success in COVID-19 patients


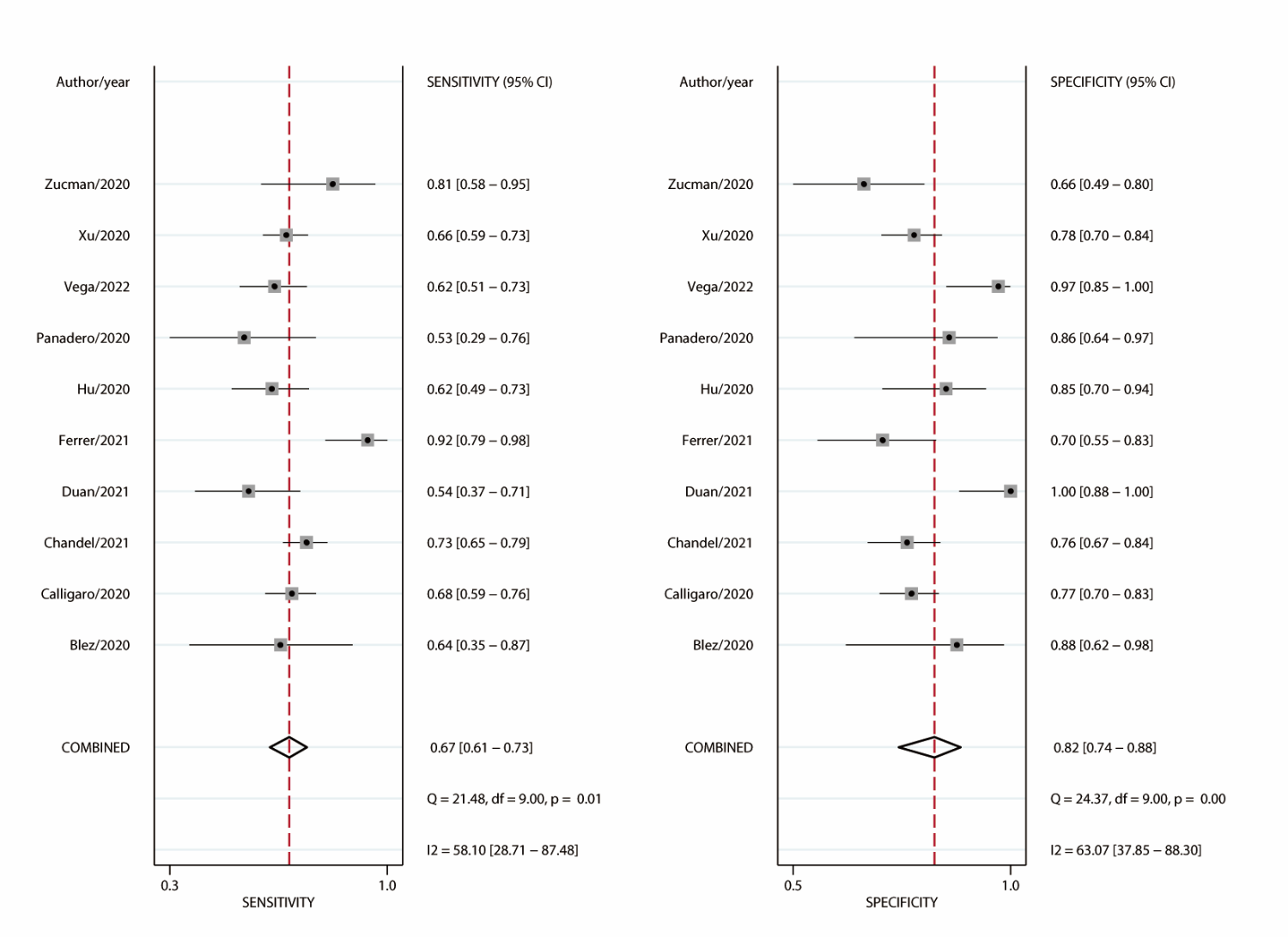


# Figure S8. HSROC of the ROX index for the prediction of HFNC success in COVID-19 patients


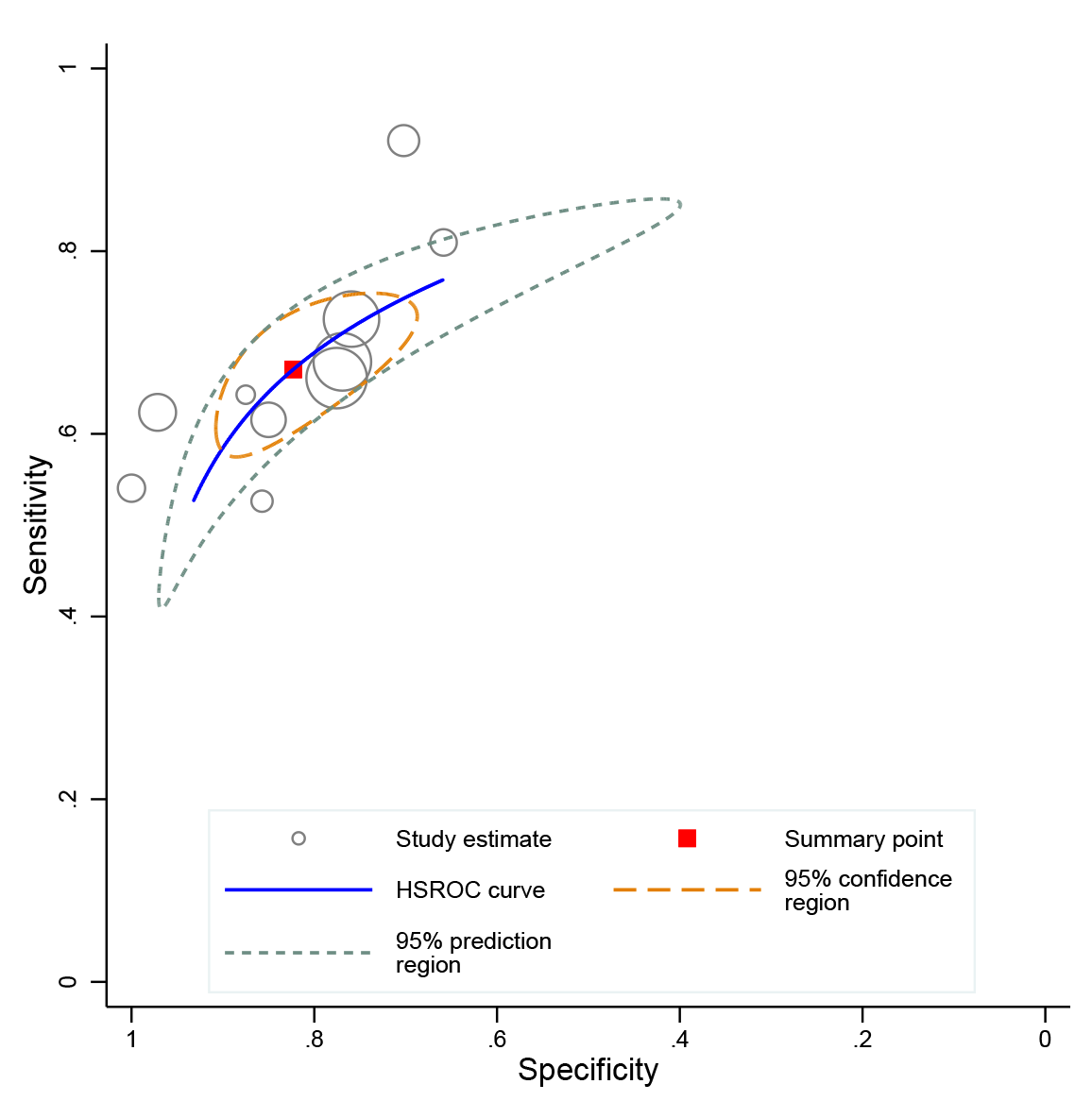


The area under the hierarchical summary receiver operating curve was 0.79 (95% CI: 0.75 to 0.82). The size of the circles indicates the weight of each individual study.
